# Supplementary material for: TMEM25 inhibits monomeric EGFR-mediated STAT3 activation in basal state to suppress triple-negative breast cancer progression
Source: Nat Commun. 2023 Apr 24;14:2342. doi: 10.1038/s41467-023-38115-2 (PMC10126118; doi:10.1038/s41467-023-38115-2)
Supplement: Supplementary file 1 — Supplementary Information [file 41467_2023_38115_MOESM1_ESM.pdf]

Supplementary Information

TMEM25 inhibits monomeric EGFR-mediated STAT3 activation in basal state to suppress triple-negative breast cancer progression

This file includes:

Supplementary Figures 1-10

Supplementary Table 1

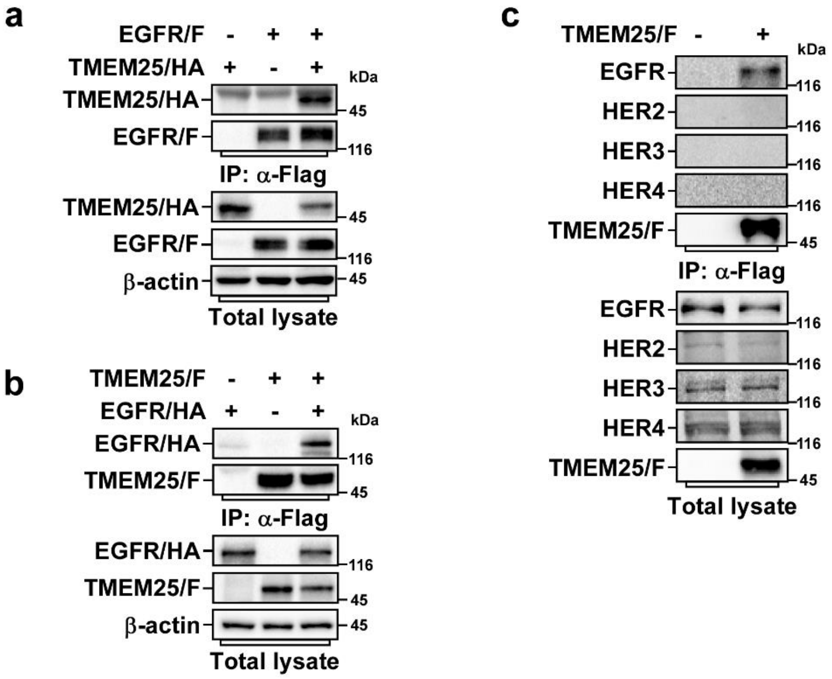

Supplementary Fig. 1. TMEM25 interacts with EGFR

a, b, Interaction between exogenous TMEM25 and EGFR. HEK293T cells transfected with combinations of C-terminal HA-tagged TMEM25 (TMEM25/HA) and C-terminal Flag-tagged EGFR (EGFR/F) (**a**), or C-terminal HA-tagged EGFR (EGFR/HA) and C-terminal Flag-tagged TMEM25 (TMEM25/F) (**b**) were subjected to anti-Flag immunoprecipitation (IP) followed by immunoblotting assay. **c**, Exogenous TMEM25 specifically interacts with endogenous EGFR but not HER2/3/4. MCF7 cells transfected with TMEM25/F were subjected to anti-Flag IP followed by immunoblotting assay to detect associated endogenous HER proteins. The blotting experiments are representative of at least three biologically independent replicates (**a**-**c**). Source data are provided as a Source Data file.

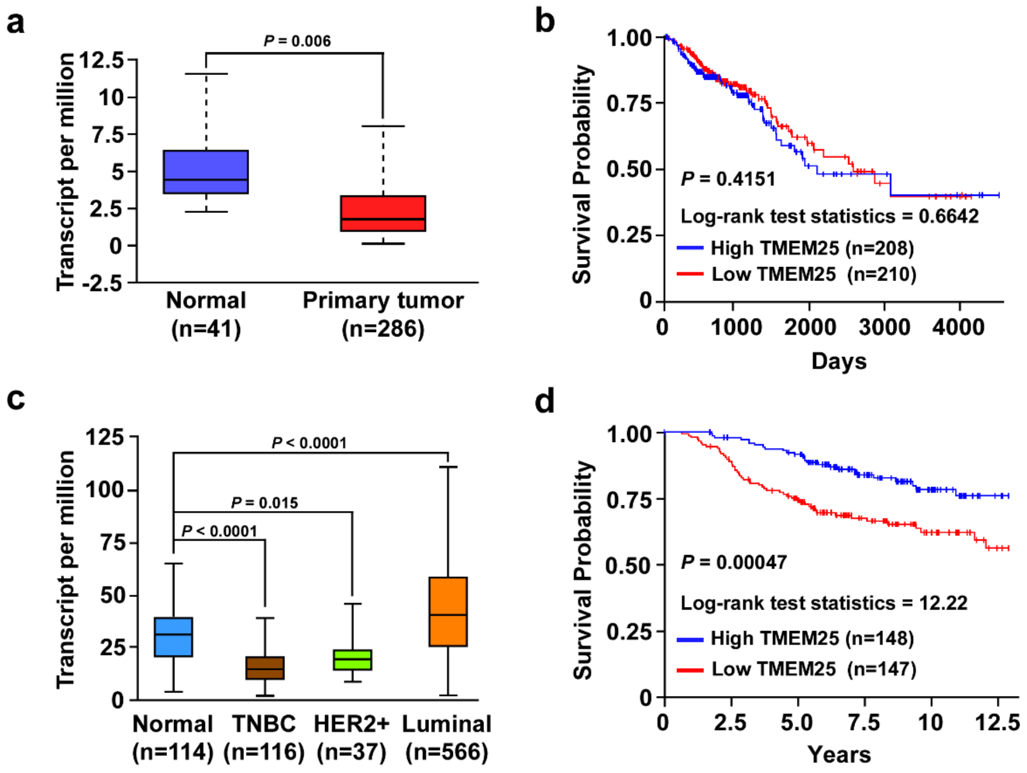

Supplementary Fig. 2. Expression of TMEM25 in colorectal and breast cancer patients

a, The mRNA levels of TMEM25 in colorectal cancers. The mRNA levels of TMEM25 in colorectal cancer samples were obtained and plotted using the UALCAN platform (http://ualcan.path.uab.edu/analysis.html). Normal (n=41), minima: 2.286, maxima: 11.586, and percentile 75%: 6.409, 50%: 4.428, 25%: 3.491. Primary tumor (n=286), minima: 0.118, maxima: 8.035, and percentile 75%: 3.342, 50%: 1.8, 25%: 0.972. P value was calculated using a Welch's T-test. **b**, Kaplan-Meier survival curves of colorectal cancer patients stratified by TMEM25 mRNA levels. Data were generated by the UCSC Xena platform. *P* value was determined by log-rank (Mantel-Cox) test, 95% confidence interval of ratio. **c**, The mRNA levels of TMEM25 in breast cancers. The mRNA levels of TMEM25 in different subtypes of breast cancer were obtained and plotted using the UALCAN platform (http://ualcan.path.uab.edu/analysis.html). Normal (n=114), minima: 4.096, maxima: 64.928, and percentile 75%: 38.777, 50%: 31.274, 25%: 20.419. TNBC (n=116), minima: 1.808,

maxima: 39.422, and percentile 75%: 20.145, 50%: 14.497, 25%: 9.578. HER2+ (n=37), minima: 8.683, maxima: 45.691, and percentile 75%: 23.497, 50%: 18.904, 25%: 14.087. Luminal (n=566), minima: 1.941, maxima: 111.598, and percentile 75%: 58.212, 50%: 40.568, 25%: 25.796. Welch's T-test was used to estimate the significance of differences in expression levels between normal and tumor subgroups. **d**, Kaplan-Meier survival curves of breast cancer patients stratified by TMEM25 mRNA levels. Data were generated by the UCSC Xena platform. *P* value was determined by log-rank (Mantel-Cox) test, 95% confidence interval of ratio.

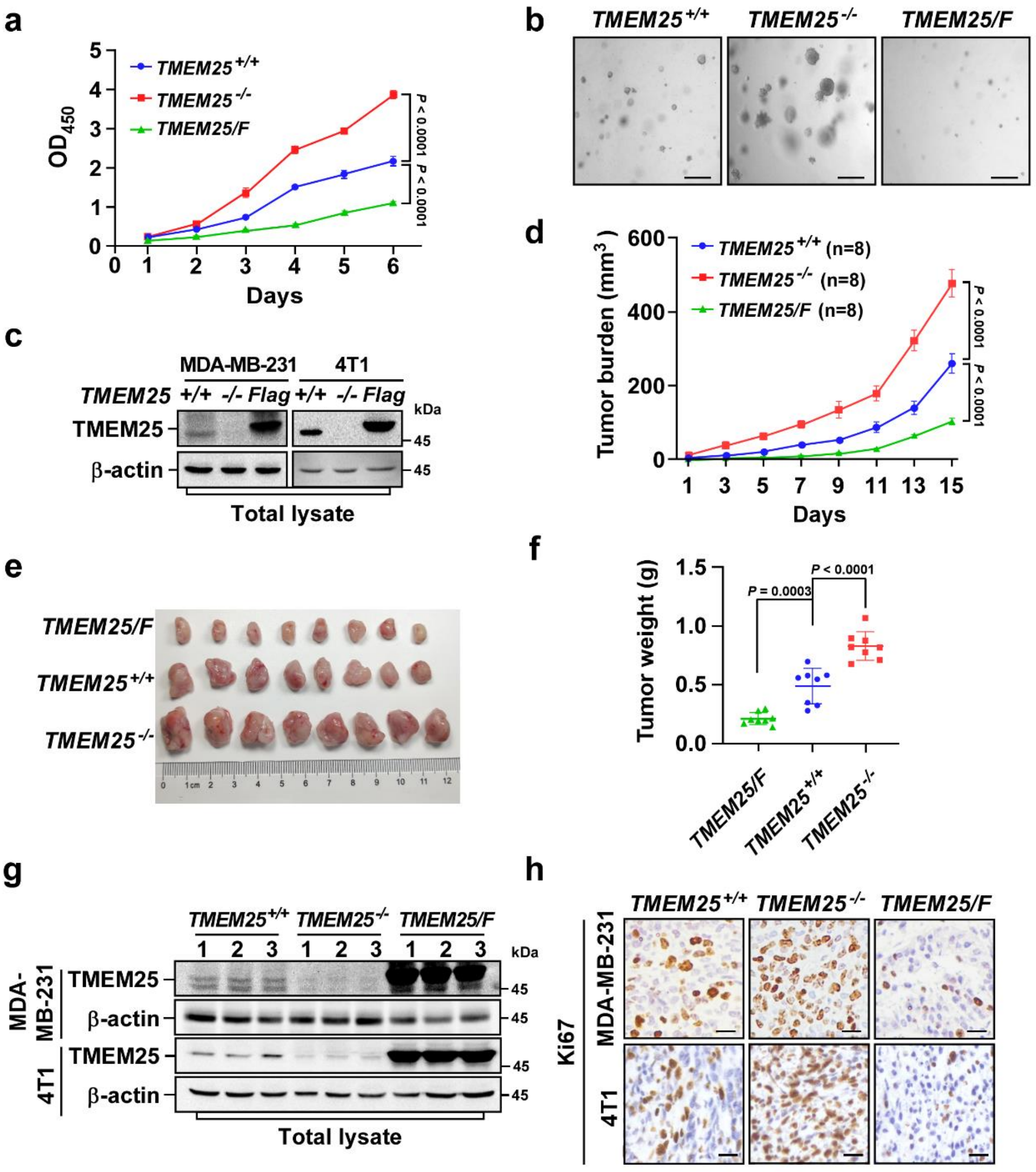

Supplementary Fig. 3. TMEM25 inhibits growth of mouse TNBC 4T1 cells *in vitro* and *in vivo*

a, Effect of TMEM25 on growth of 4T1 cells. Cell viability of wild-type (*TMEM25*$^{+/+}$), *TMEM25* knockout (*TMEM25*$^{-/-}$), and Flag-tagged TMEM25 overexpressing (*TMEM25/F*) 4T1 cells were determined using CCK-8 assay. Data were presented as mean ± SEM of three individual experiments. *P* values were determined by two-way ANOVA followed by Tukey test. **b**, Effect of TMEM25 on colony formation of 4T1 cells. *TMEM25*$^{+/+}$, *TMEM25*$^{-/-}$, and

TMEM25/F 4T1 cells were subjected to soft agar assay. Scale bar indicates 100 μm. At least three biological replicates were performed for each condition. **c**, Expression levels of TMEM25 in cells grown *in vitro*. Total lysates of MDA-MB-231 or 4T1 cells were subjected to immunoblotting assay to examine the expression levels of TMEM25. **d-f**, TMEM25 suppresses tumor growth of 4T1 cells in BALB/c mice. $TMEM25^{+/+}$, $TMEM25^{-/-}$, or *TMEM25/F* 4T1 cells were orthotopically injected into the mammary fat pad of BALB/c mice to form breast tumor. The volumes of tumors were measured and plotted as mean ± SEM of 8 mice per group. *P* values were determined by two-way ANOVA followed by Tukey test (**d**). Mice were sacrificed 15 days after tumor formation to obtain the tumors (**e**). The tumors were weighted and plotted as mean ± SEM of 8 mice per group. *P* values were determined by one-way ANOVA followed by Tukey test (**f**). **g,** Expression levels of TMEM25 in transplant TNBC tumors. Representative tumors were subjected to immunoblotting assay to examine the expression levels of TMEM25. **h,** TMEM25 decreases Ki67 expression in transplanted tumors. Representative tumors were subjected to immunohistochemistry (IHC) assay to examine the Ki67 levels. Scale bar indicates 100 μm. The blotting experiments are representative of at least three biologically independent replicates (**c, g**). Source data are provided as a Source Data file.

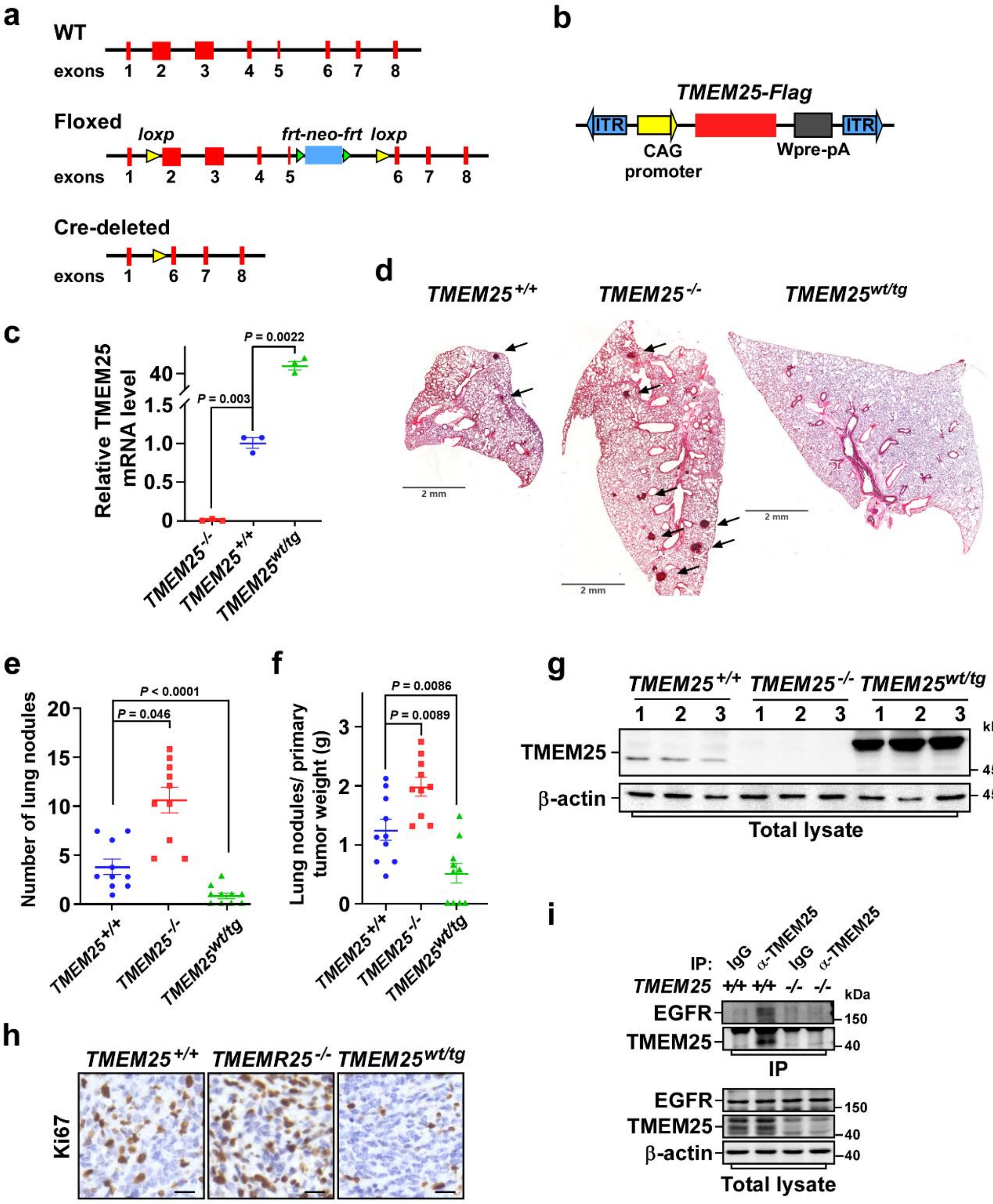

Supplementary Fig. 4. Construction of TMEM25 knockout and transgenic mice

a, A diagram illustrating generation of *TMEM25*$^{-/-}$ mice. The red boxes represent exons. Deletion of exon 2-5 with Cre recombinase results in a frame-shift mutation which ablates TMEM25 expression. **b**, A diagram illustrating generation of *TMEM25*$^{wt/tg}$ mice. The red boxes represent TMEM25 cDNA with Flag tag at C-terminal. The transposase TMGI system were used to generate transgenic mice. **c**, Relative TMEM25 mRNA levels in *TMEM25*$^{+/+}$,

TMEM25$^{-/-}$ and *TMEM25*$^{wt/tg}$ mice. The mRNA levels of TMEM25 in *TMEM25*$^{+/+}$, *TMEM25*$^{-/-}$ and *TMEM25*$^{wt/tg}$ *MMTV-PyMT* mice were determined by real-time quantitative PCR using GAPDH as an internal control. Data are presented as mean ± SEM of three independent experiments. *P* values were determined by one-way ANOVA followed by Tukey test. **d-f**, TMEM25 inhibits lung metastasis of breast cancer cells in *MMTV-PyMT* mice. Representative images of hematoxylin and eosin (H&E)-stained lung sections were presented. Arrows indicate lung metastasis nodules. Scale bar indicates 2 mm (**d**). The numbers of lung metastasis nodules were counted and presented as mean ± SEM of 10 mice per group. *P* values were determined by one-way ANOVA followed by Tukey test (**e**). Normalization of lung metastases to tumor burden. The lung metastasis nodules numbers to primary tumor weights were calculated and plotted as mean ± SEM (**f**). **g,** Expression levels of TMEM25 in spontaneous TNBC tumors in *MMTV-PyMT* mice. Representative tumors spontaneously formed in *MMTV-PyMT* mice were treated with collagenase IV to remove extracellular matrix and the collected cells were subjected to immunoblotting assay to examine the expression levels of TMEM25. **h,** TMEM25 decreases Ki67 expression in spontaneous TNBC tumors in *MMTV-PyMT* mice. Representative tumors were subjected to immunohistochemistry (IHC) assay to examine the Ki67 levels. Scale bar indicates 100 μm. **i,** TMEM25 interacts with EGFR in spontaneous tumor formed in *MMTV-PyMT* mice. Cells collected from tumors formed in *MMTV-PyMT* mice were subjected to anti-TMEM25 IP followed by immunoblotting to detect associated EGFR. The blotting experiments are representative of at least three biologically independent replicates (**g, i**). Source data are provided as a Source Data file.

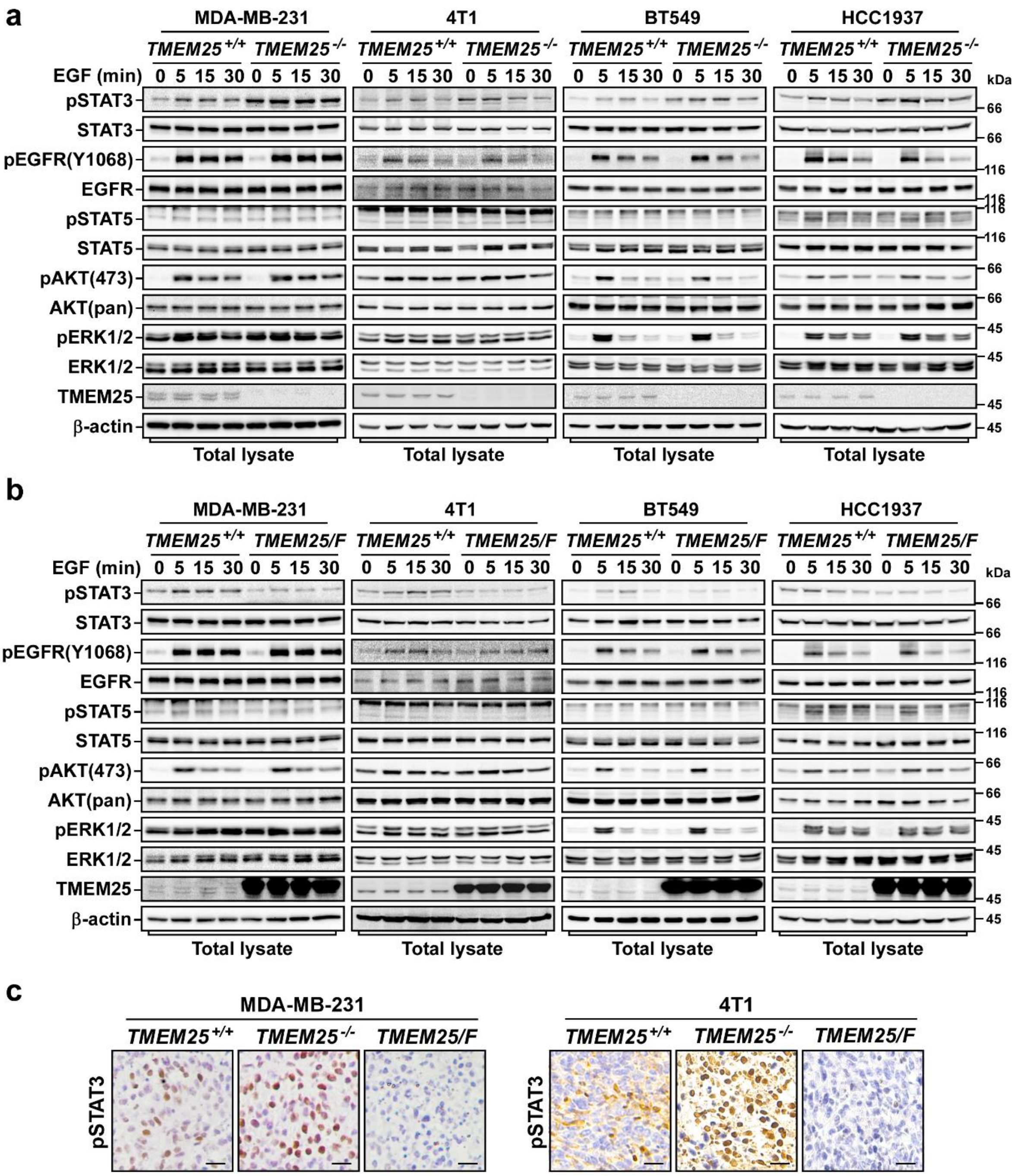

Supplementary Fig. 5. TMEM25 suppresses STAT3 activation in TNBC cells

a, Knockout of *TMEM25* induces phosphorylation of STAT3 in TNBC cells. *TMEM25*$^{+/+}$ or *TMEM25*$^{-/-}$ MDA-MB-231, 4T1, BT549, or HCC1937 cells were serum-starved overnight and then treated with 100 ng/ml EGF for the indicated time before subjected to immunoblotting assay. **b**, Overexpression of TMEM25 inhibits EGF treatment-induced phosphorylation of

STAT3 in TNBC cells. *TMEM25*$^{+/+}$ or Flag-tagged TMEM25 overexpressing (*TMEM25/F*) MDA-MB-231, 4T1, BT549, or HCC1937 cells were treated and analyzed as in (**a**). **c**, TMEM25 inhibits STAT3 phosphorylation in transplanted TNBC tumors. Representative tumors formed by MDA-MB-231 or 4T1 cells were subjected to IHC assay to examine the levels of phosphorylated STAT3 as indicated. Scale bar indicates 100 μm. The blotting experiments are representative of at least three biologically independent replicates (**a, b**). Source data are provided as a Source Data file.

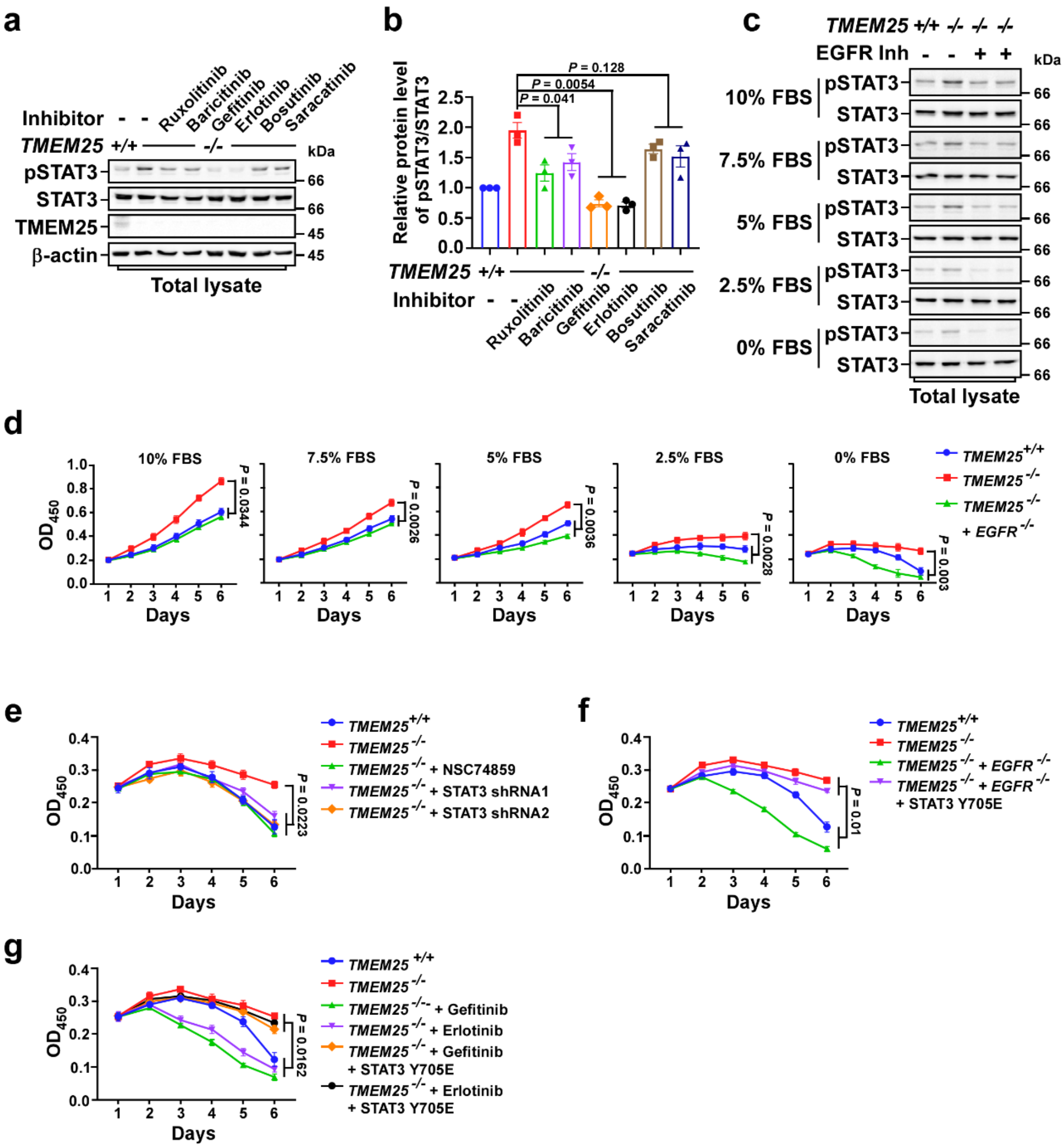

Supplementary Fig. 6. EGFR is required for TMEM25 knockout-induced STAT3 phosphorylation

a, b, EGFR activity is critical for *TMEM25* knockout-promoted STAT3 phosphorylation. After overnight serum starvation, *TMEM25*$^{-/-}$ MDA-MB-231 cells were treated with/without JAK1/2 inhibitor Ruxolitinib (0.5 μM, 1 h) or Baricitinib (2.5 μM, 24 h), EGFR inhibitor Gefitinib (20 μM, 24 h) or Erlotinib (20 μM, 4 h), or SRC inhibitor Bosutinib (10 μM, 1 h) or Saracatinib (10 μM, 24 h) as indicated before subjected to immunoblotting assay (**a**). The relative ratios of

pSTAT3 to total STAT3 (pSTAT3/STAT3) were determined and plotted as mean ± SEM of 3 independent experiments. *P* values were determined by one-way ANOVA followed by Tukey test (**b**). **c**, EGFR activity is required for *TMEM25* knockout-induced STAT3 phosphorylation in different serum conditions. Wild-type or *TMEM25*$^{-/-}$ MDA-MB-231 cells grown in indicated concentrations of FBS were treated with/without EGFR inhibitor Gefitinib (20 μM, 24 h) or Erlotinib (20 μM, 4 h) and then subjected to immunoblotting assay. **d**, EGFR is required for *TMEM25* knockout-promoted cell proliferation and survival. MDA-MB-231 cells (*TMEM25*$^{+/+}$, *TMEM25*$^{-/-}$, *or TMEM25*$^{-/-}$*EGFR*$^{-/-}$) were cultured in indicated concentrations of FBS and cell viabilities were determined and plotted as mean ± SEM of 3 independent experiments. *P* values were determined by one-way ANOVA followed by Tukey test. **e**, STAT3 is required for *TMEM25* knockout-promoted cell survival. MDA-MB-231 cells (*TMEM25*$^{+/+}$ *or TMEM25*$^{-/-}$) treated with STAT3 inhibitor NSC74859 (50 μM) or transduced with lentivirus encoding shRNAs against STAT3 (sh-STAT3-1/2) were cultured without FBS and cell viabilities were determined and plotted as mean ± SEM of 3 independent experiments. *P* values were determined by one-way ANOVA followed by Tukey test. **f**, **g**, Constitutively active STAT3 mutant is able to rescue *TMEM25* knockout-promoted cell survival in the absence of EGFR. *TMEM25*$^{-/-}$ MDA-MB-231 cells with further knockout of EGFR (**f**), or treatment of EGFR inhibitor Gefitinib (20 μM) or Erlotinib (20 μM) (**g**) were transduced with/without lentivirus encoding STAT3-Y705E as indicated and cultured without FBS. Cell viabilities were determined and plotted as mean ± SEM of 3 independent experiments. *P* values were determined by one-way ANOVA followed by Tukey test. The blotting experiments are representative of at least three biologically independent replicates (**a, c**). Source data are provided as a Source Data file.

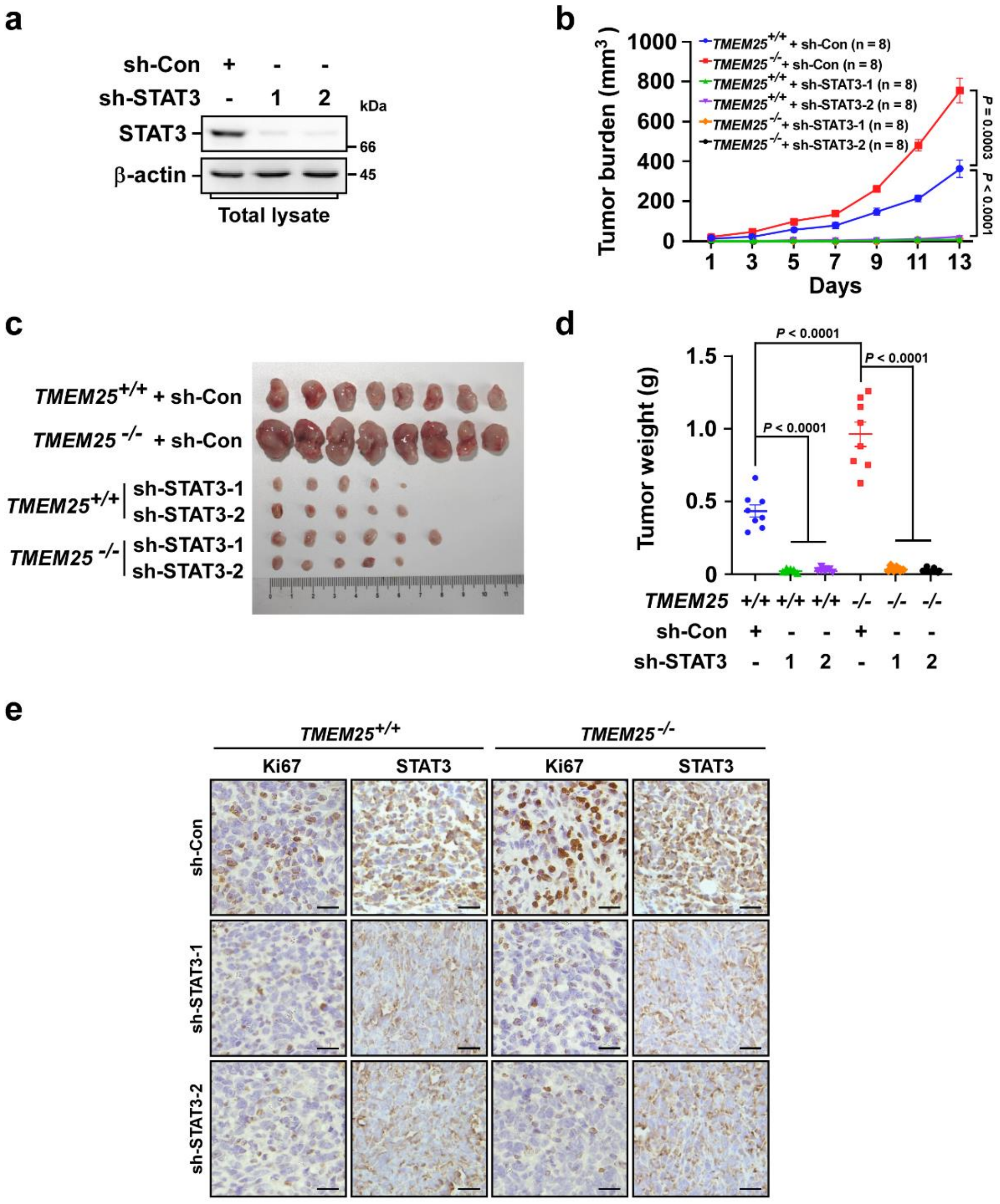

Supplementary Fig. 7. STAT3 is required for *TMEM25* knockout-promoted TNBC growth.

a, Knockdown of STAT3 in MDA-MB-231 cells. MDA-MB-231 cells transduced with lentivirus encoding control shRNA (sh-Con) or shRNAs against STAT3 (sh-STAT3-1 and sh-STAT3-2) were subjected to immunoblotting assay to examine the knockdown efficiency of STAT3. The blotting experiments are representative of at least three biologically independent

replicates. **b-d**, STAT3 knockdown suppresses tumor growth of both *TMEM25*$^{+/+}$ and *TMEM25*$^{-/-}$ MDA-MB-231 cells in nude mice. *TMEM25*$^{+/+}$ and *TMEM25*$^{-/-}$ MDA-MB-231 cells transduced with lentivirus encoding sh-Con, sh-STAT3-1 or sh-STAT3-2 were orthotopically injected into the mammary fat pad of nude mice. The volumes of tumors were measured and plotted as mean ± SEM of 8 mice per group. *P* values were determined by two-way ANOVA followed by Tukey test (**b**). Mice were sacrificed 13 days after tumor formation to obtain the tumors (**c**). The tumors were weighted and plotted as mean ± SEM of 8 mice per group. *P* values were determined by one-way ANOVA followed by Tukey test (**d**). **e,** Knockdown of STAT3 decreases Ki67 expression in both *TMEM25*$^{+/+}$ and *TMEM25*$^{-/-}$ tumors. Ki67 and STAT3 expression in xenograft mouse tumors were examined by IHC assay. Scale bar indicates 100 μm. Source data are provided as a Source Data file.

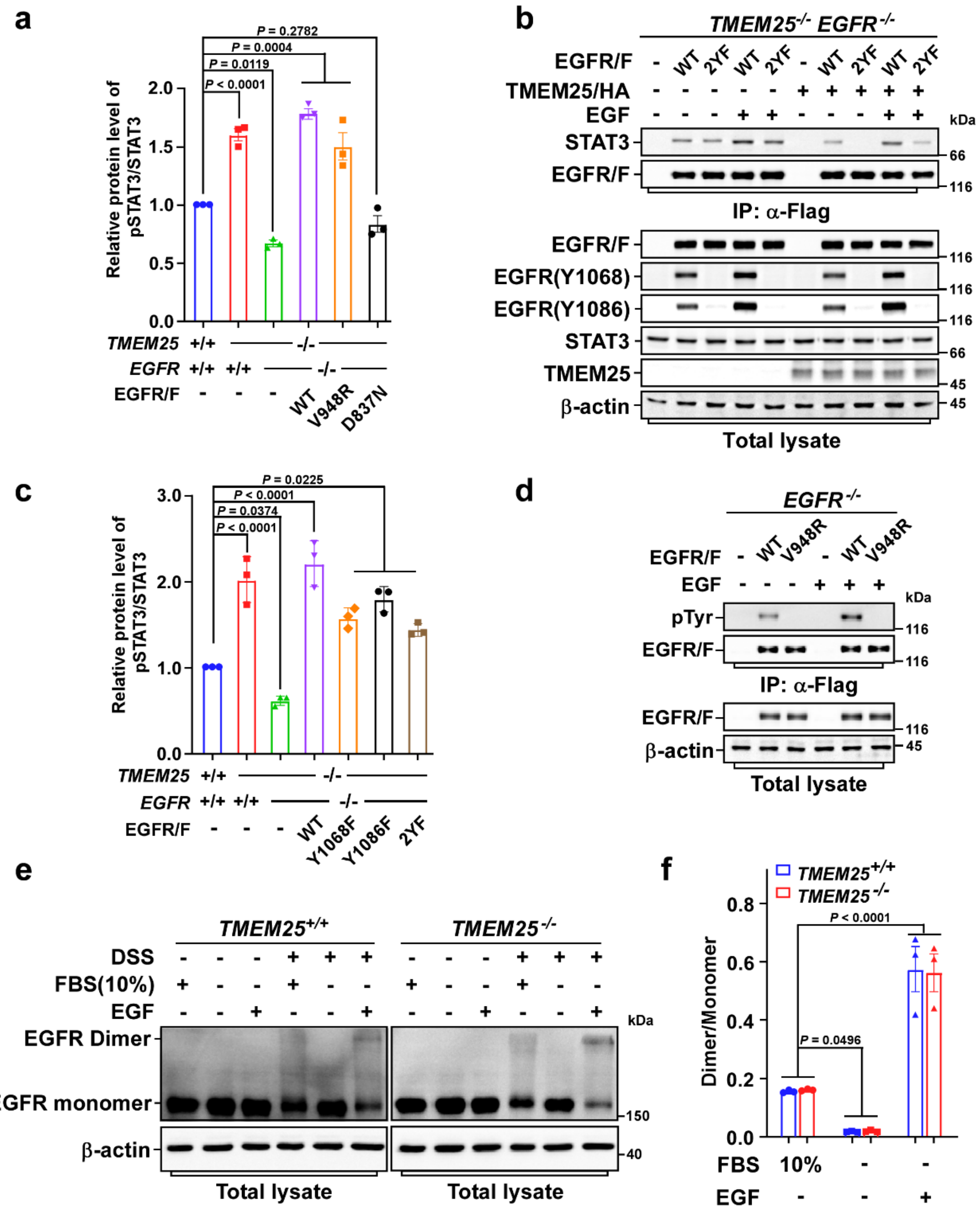

Supplementary Fig. 8. Monomeric EGFR is able to phosphorylate STAT3 in the absence of TMEM25

a, EGFR-V948R phosphorylates STAT3 with a similar efficiency as wild-type EGFR. *TMEM25*$^{-/-}$*EGFR*$^{-/-}$ MDA-MB-231 cells transduced with/without lentivirus encoding EGFR/F (WT, V948R, or D837N) as indicated were serum starved overnight before subjected to immunoblotting assay to determine the pSTAT3/STAT3 ratios. Quantitative analysis of the

results is presented relative to the pSTAT3/STAT3 ratio in wild-type MDA-MB-231 cells as mean ± SEM of 3 independent experiments. *P* values were determined by one-way ANOVA followed by Tukey test. **b**, TMEM25 has a stronger effect on blocking STAT3 and EGFR-Y1068,1086F (2YF) mutant interaction than STAT3 and wild-type EGFR interaction. *TMEM25*$^{-/-}$*EGFR*$^{-/-}$ MDA-MB-231 cells transduced with indicated combinations of lentivirus encoding EGFR/F (WT or 2YF) and TMEM25/HA were treated with/without EGF (100 ng/ml, 15 min) and then subjected to anti-Flag IP followed by immunoblotting assay to detect associated endogenous STAT3. **c**, Disruption of phosphorylation of Y1068 or Y1086 of EGFR partially affects EGFR-mediated phosphorylation of STAT3 in the absence of TMEM25. Same as in (**a**) except EGFR-Y1068F, -Y1086F, or -2YF instead of -V948R or -D837N were used. *P* values were determined by one-way ANOVA followed by Tukey test. **d**, Tyrosine phosphorylation in EGFR-V948R. *EGFR*$^{-/-}$ MDA-MB-231 cells transduced with lentivirus encoding EGFR/F (WT or V948R) were treated with/without EGF (100 ng/ml, 5 min) and then subjected to anti-Flag IP followed by immunoblotting assay to detect the global tyrosine phosphorylation of EGFR using pY20 antibody. **e, f,** Dimer formation of EGFR under different conditions in *TMEM25*$^{+/+}$ and *TMEM25*$^{-/-}$ cells. MDA-MB-231 cells cultured in 10% FBS medium or FBS-deprived medium overnight treated with/without EGF (100 ng/ml, 5 min) as indicated were treated with/without 1 mM crosslinker disuccinimidyl suberate (DSS) for 1 h at 4°C before subjected to immunoblotting assay to examine the dimer formation of EGFR (**e**). The amounts of EGFR monomer and dimer were quantified and the ratios of dimer to monomer were plotted as mean ± SEM of 3 independent experiments. *P* values were determined by one-way ANOVA followed by Tukey test (**f**). The blotting experiments are representative of at least three biologically independent replicates (**b, d, e**). Source data are provided as a Source Data file.

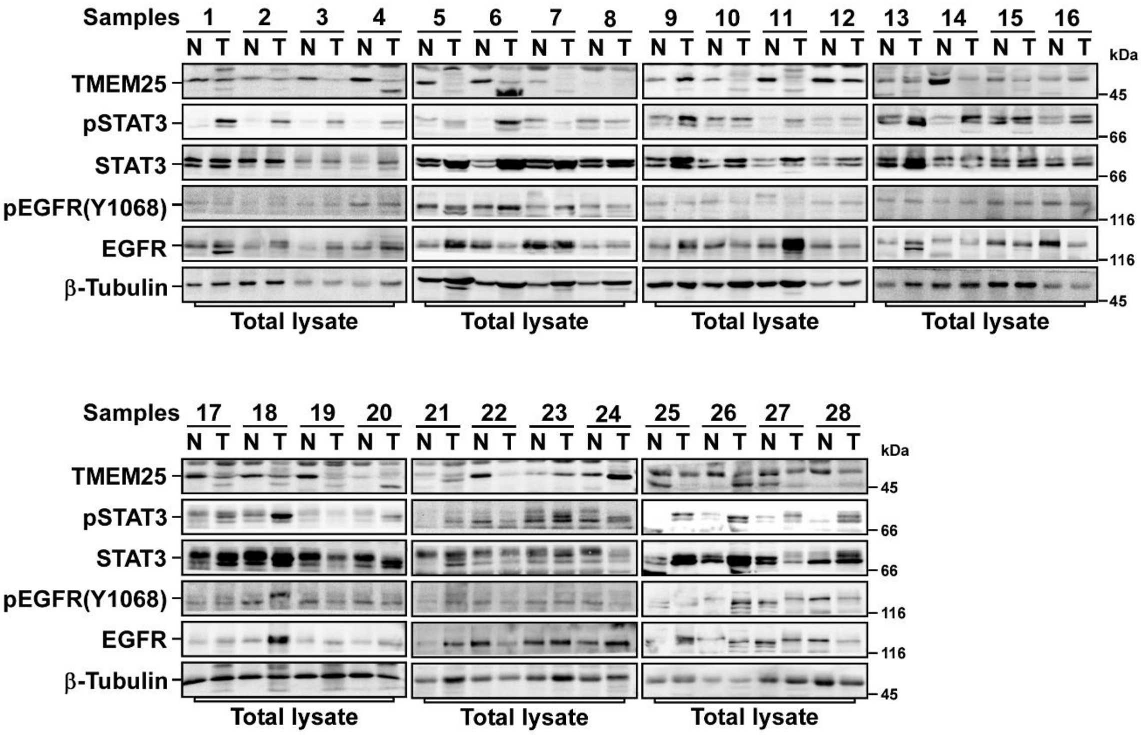

Supplementary Fig. 9. TMEM25 expression in TNBC clinical samples

Expression levels of TMEM25 and phosphorylation levels of STAT3 in human TNBC specimens. The extracts from tumor tissues (T) and their matched surrounding normal tissues (N) were subjected to immunoblotting assay. The blotting experiments are representative of three biologically independent replicates. Source data are provided as a Source Data file.

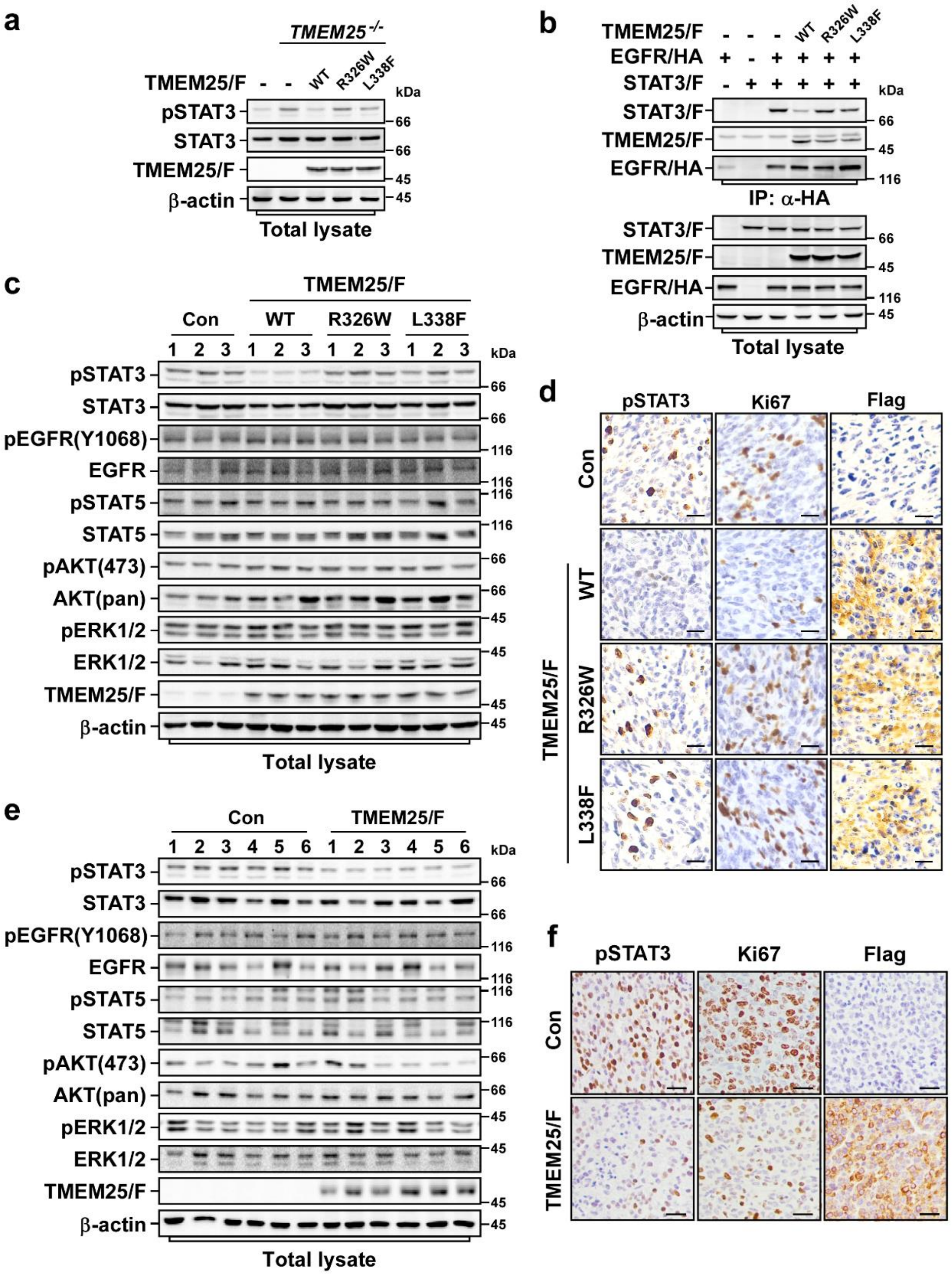

Supplementary Fig. 10. Effects of clinical mutations of TMEM25 on inhibiting phosphorylation of STAT3 and growth of TNBC cells

a, R326W or L338F mutation disrupts the inhibitory effect of TMEM25 on STAT3 phosphorylation. *TMEM25*$^{-/-}$ MDA-MB-231 cells transduced with lentivirus encoding

TMEM25 (WT, R326W, or L338F) were subjected to immunoblotting assay. **b,** R326W or L338F mutation disrupts the blockade effect of TMEM25 on binding of STAT3 to EGFR. HEK293T cells transduced with TMEM25/F (WT, R326W, or L338F), EGFR/HA and STAT3/F as indicated were subjected to anti-HA IP followed by immunoblotting assay to detect associated STAT3. **c, d**, Wild-type but not R326W or L338F TMEM25 inhibits STAT3 phosphorylation in the spontaneous breast tumors in *MMTV-PyMT* mice. Representative tumors from *MMTV-PyMT* mice injected with control AAV or AAV encoding TMEM25 (WT, R326W, or L338F) were subjected to immunoblotting (**c**) or IHC assay (**d**). Scale bar indicates 100 μm. **e, f,** TMEM25 inhibits STAT3 phosphorylation in the PDX mouse tumors. Tumors from PDX mice injected with control AAV or AAV encoding TMEM25 were subjected to immunoblotting (**e**) or IHC assay (**f**). Scale bar indicates 100 μm. The blotting experiments are representative of at least three biologically independent replicates (**a**-**c, e**). Source data are provided as a Source Data file.

Supplementary Table 1. The information of TNBC clinical samples in this study.

Patients	Gender	Age (years)	Patients	Gender	Age (years)
1	Female	50	15	Female	71
2	Female	57	16	Female	55
3	Female	58	17	Female	57
4	Female	45	18	Female	82
5	Female	30	19	Female	49
6	Female	60	20	Female	41
7	Female	33	21	Female	47
8	Female	43	22	Female	82
9	Female	34	23	Female	43
10	Female	48	24	Female	48
11	Female	61	25	Female	59
12	Female	66	26	Female	70
13	Female	43	27	Female	48
14	Female	59	28	Female	55

Uncropped scans of blots:

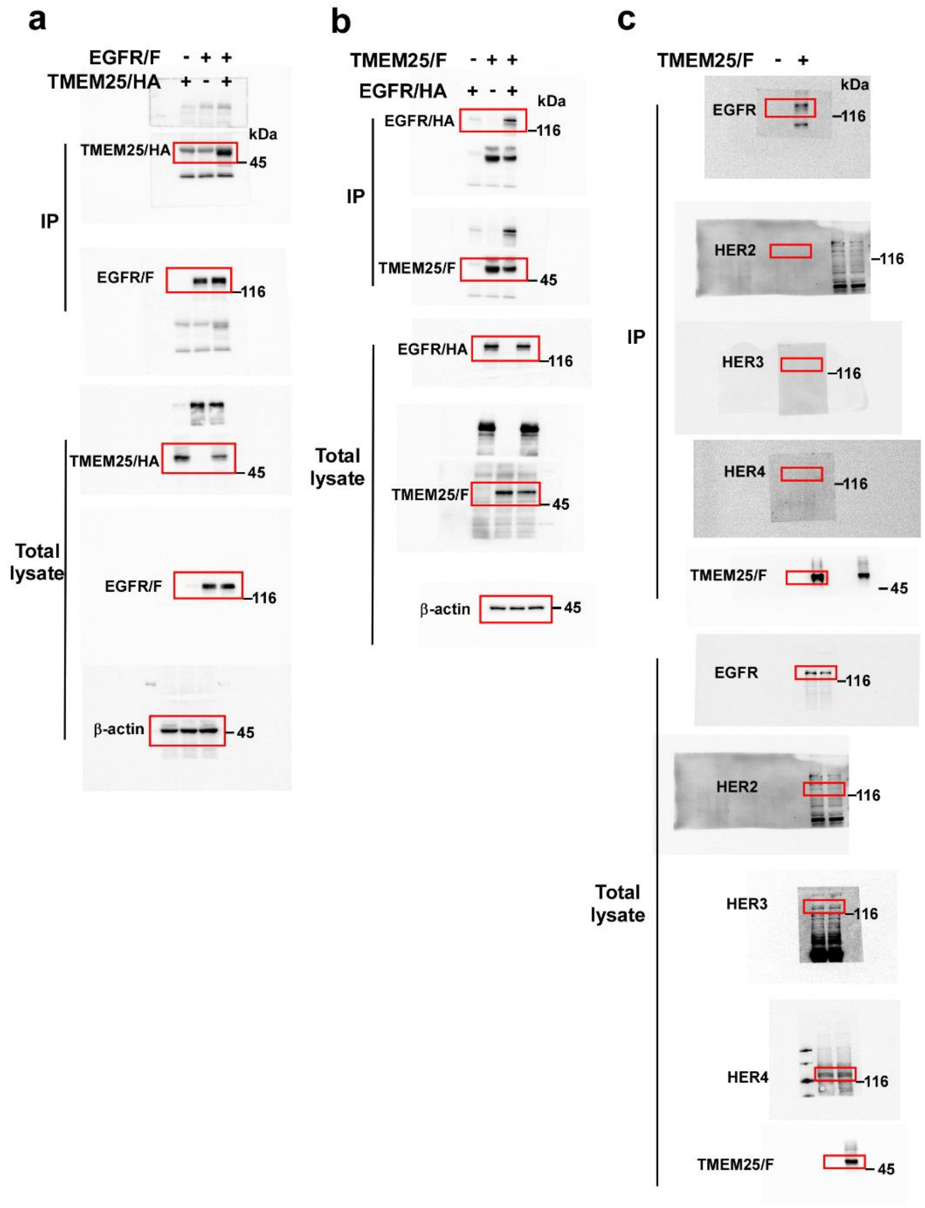

Supplementary Fig. 1

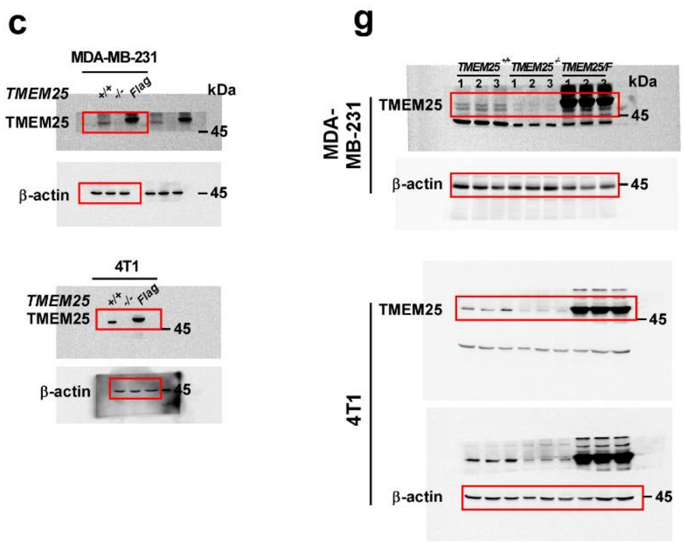

Supplementary Fig. 3

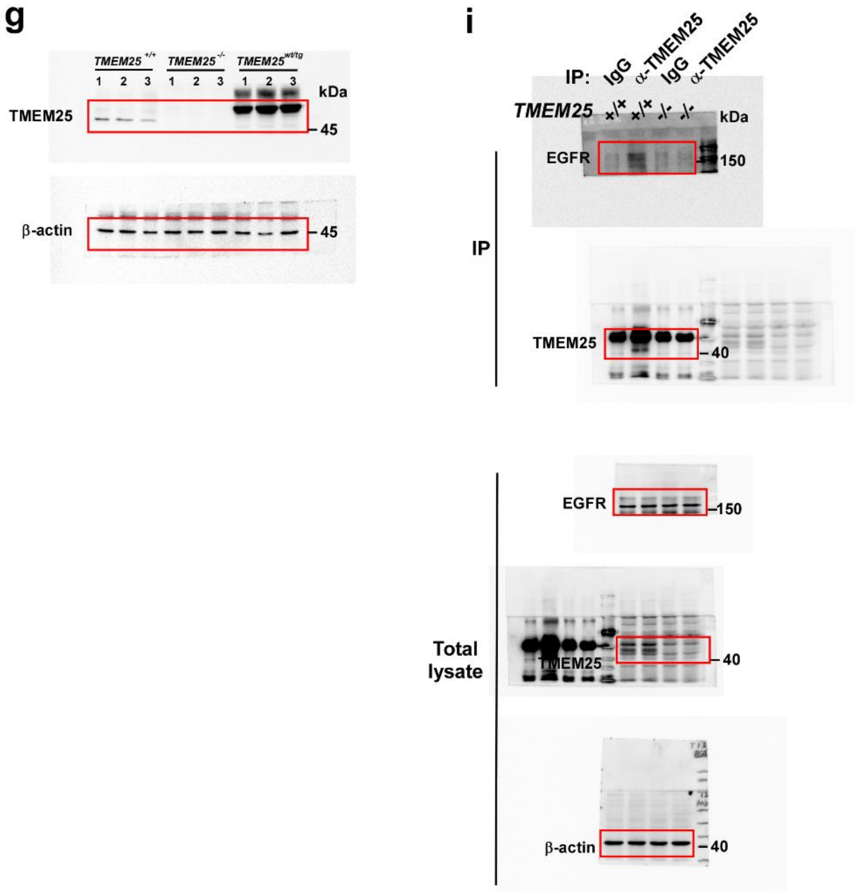

Supplementary Fig. 4

a

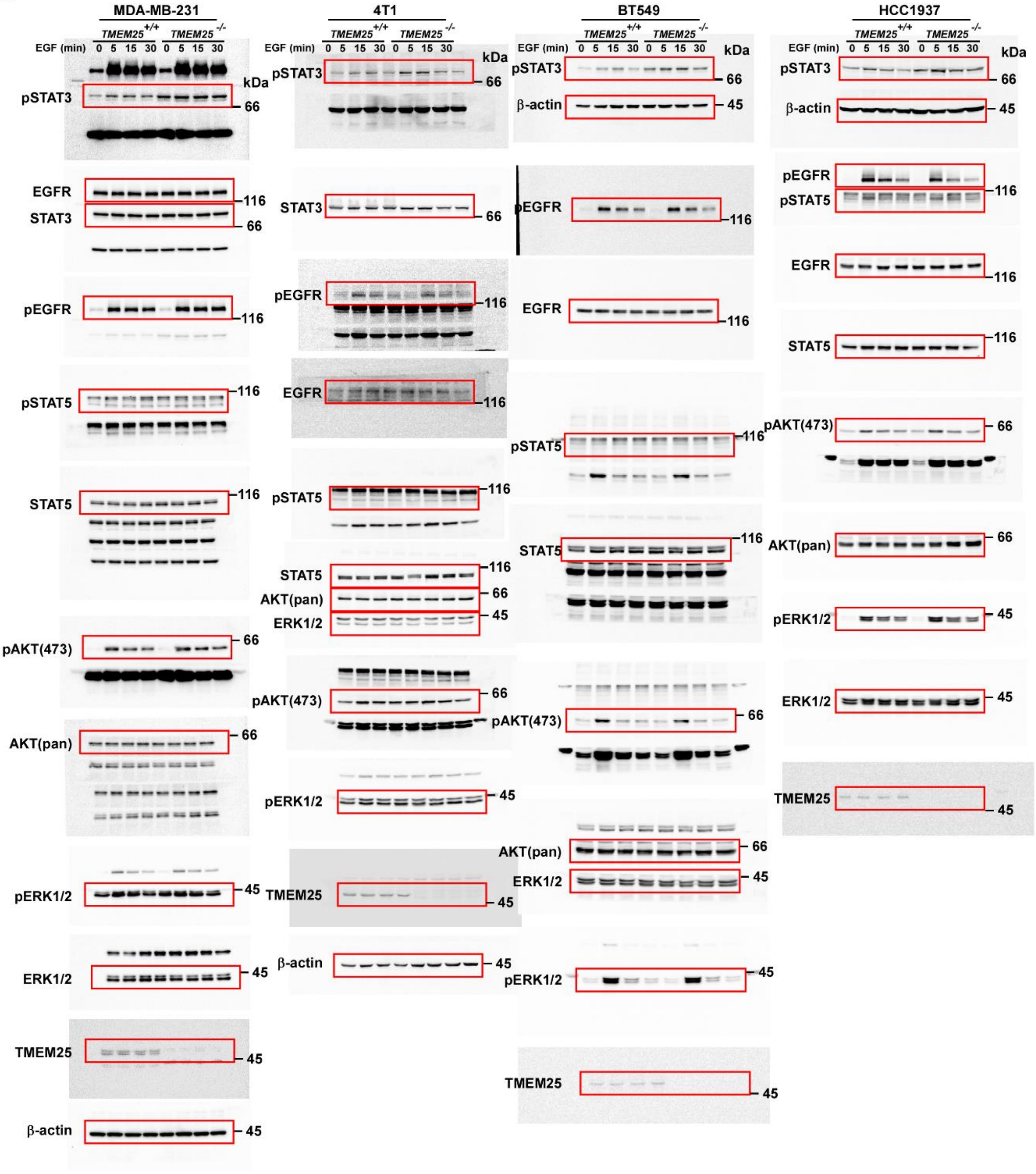
MDA-MB-231
4T1
BT549
HCC1937
TMEM25+/+
TMEM25-/-
EGF (min)
0 5 15 30 0 5 15 30
kDa
pSTAT3
EGFR
STAT3
pEGFR
pSTAT5
STAT5
pAKT(473)
AKT(pan)
pERK1/2
ERK1/2
TMEM25
β-actin
116
66
45

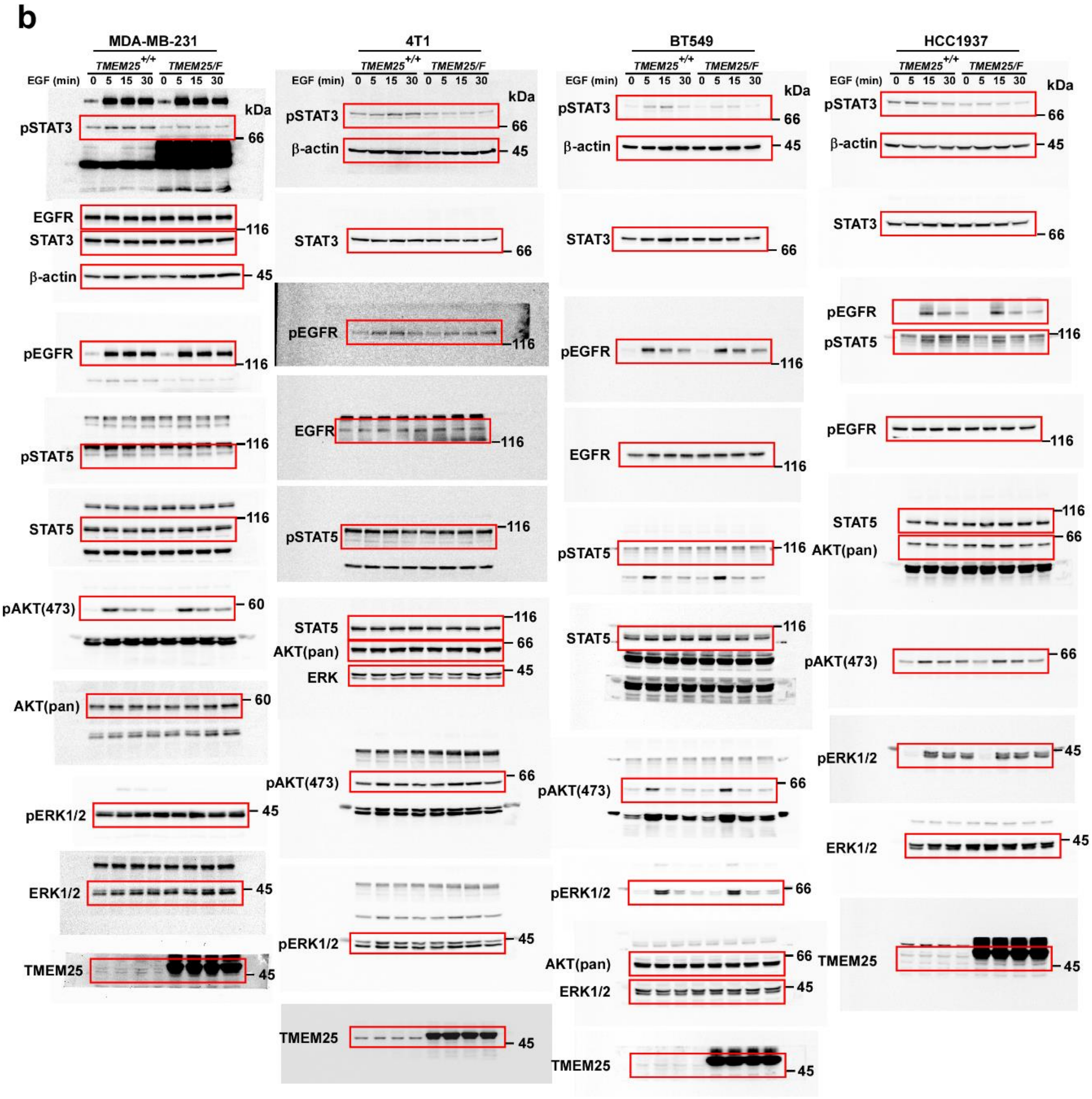

Supplementary Fig. 5

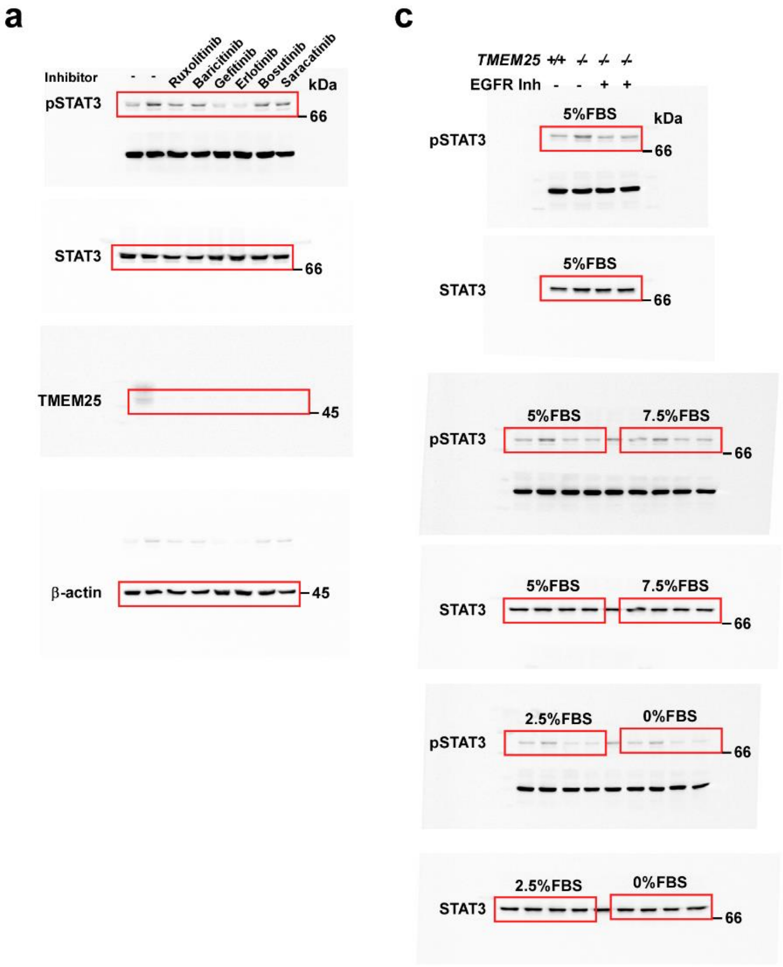

Supplementary Fig. 6

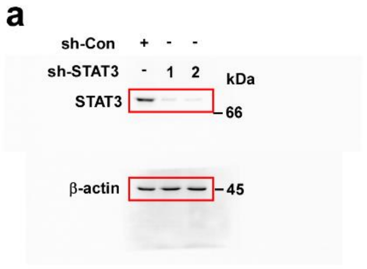

Supplementary Fig. 7

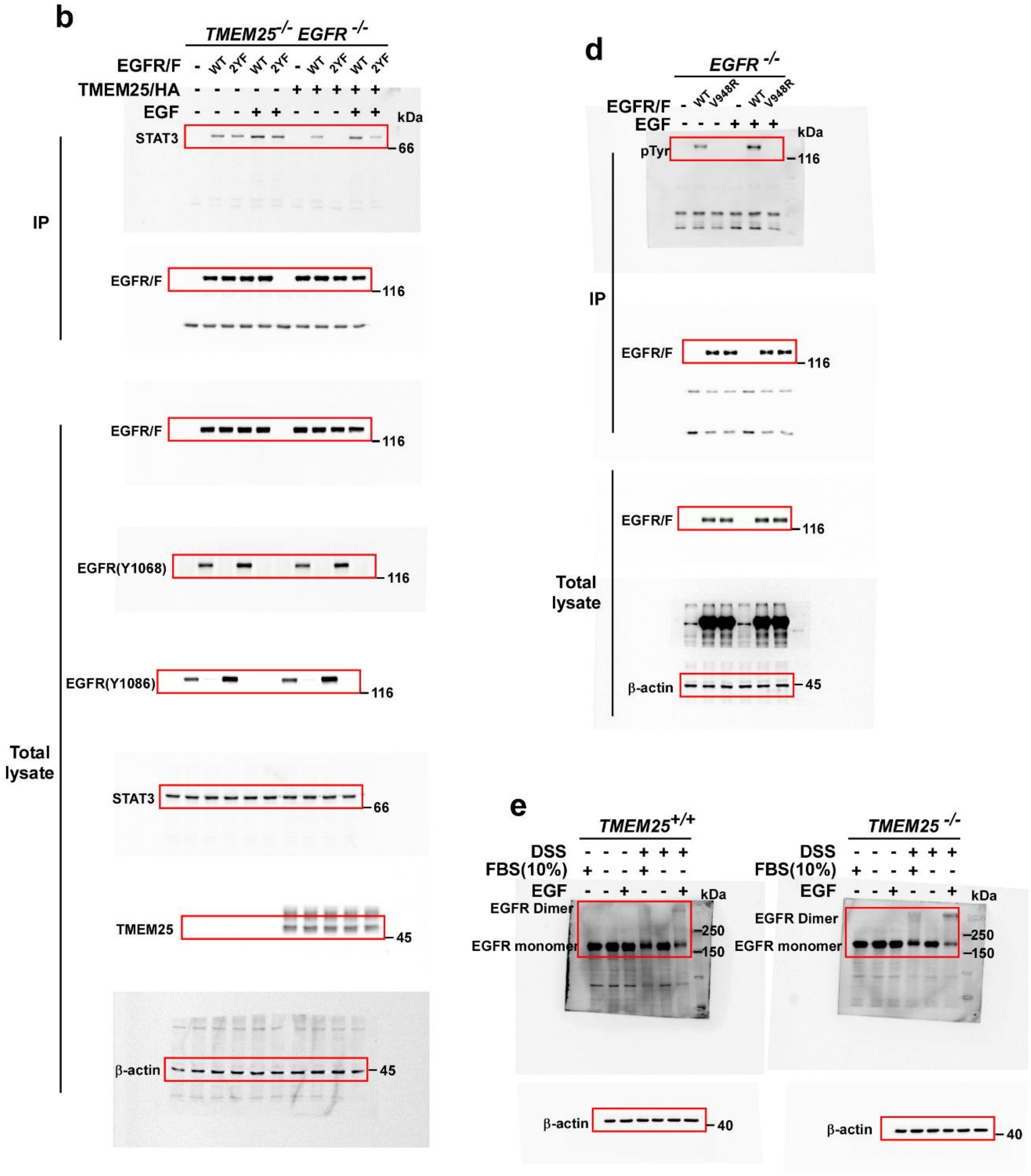

Supplementary Fig. 8

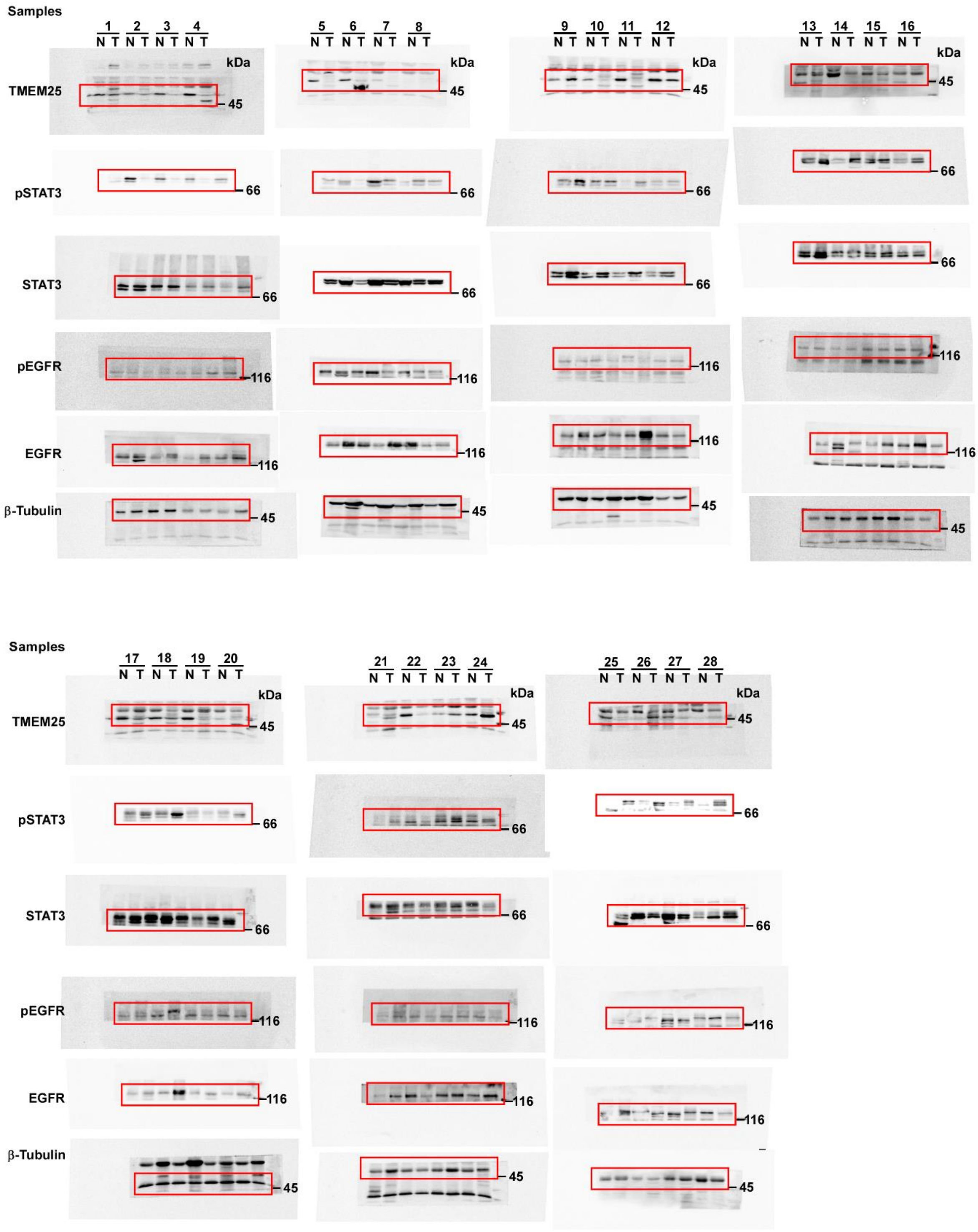

Supplementary Fig. 9

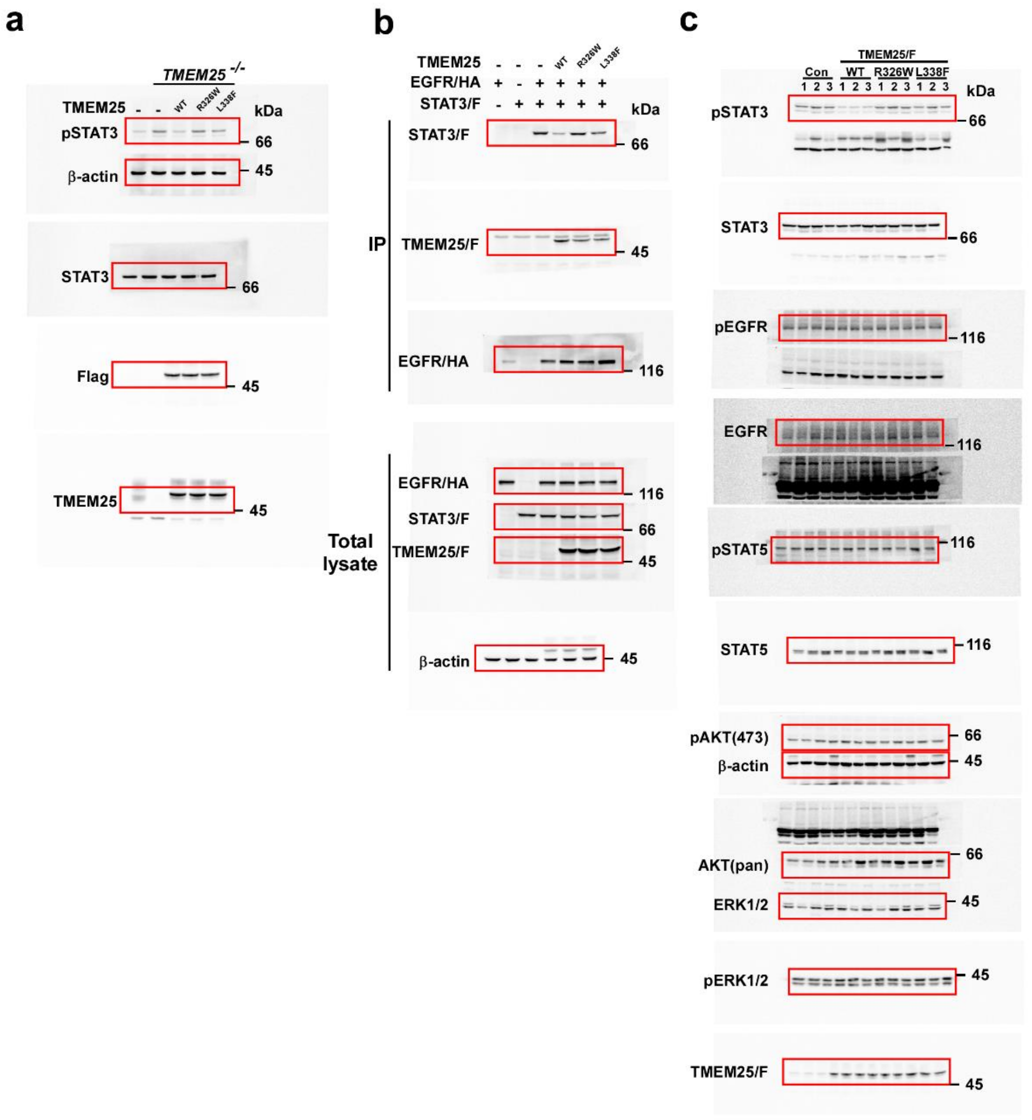
a
TMEM25 -/-
TMEM25
kDa
pSTAT3
β-actin
STAT3
Flag
TMEM25
b
TMEM25
EGFR/HA
STAT3/F
IP
TMEM25/F
Total lysate
c
TMEM25/F
Con
WT
R326W
L338F
pEGFR
EGFR
pSTAT5
STAT5
pAKT(473)
AKT(pan)
ERK1/2
pERK1/2

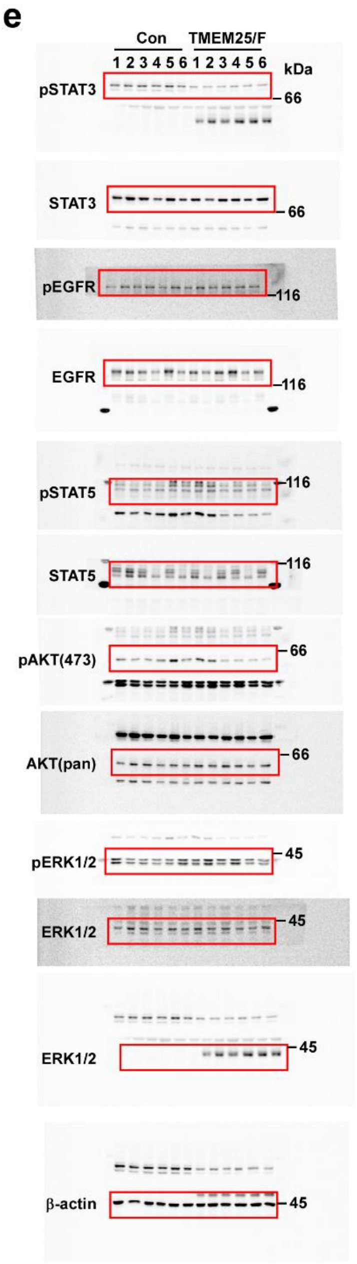

Supplementary Fig. 10
