# Supplementary material for: TMEM25 inhibits monomeric EGFR-mediated STAT3 activation in basal state to suppress triple-negative breast cancer progression
Source: Nat Commun. 2023 Apr 24;14:2342. doi: 10.1038/s41467-023-38115-2 (PMC10126118; doi:10.1038/s41467-023-38115-2)
Supplement: Supplementary file 3 — Reporting Summary [file 41467_2023_38115_MOESM3_ESM.pdf]

nature portfolio

Corresponding author(s): Guo Fu, Tong-Jin Zhao, and Hong-Rui Wang

Last updated by author(s): Mar 26, 2023

Reporting Summary

Nature Portfolio wishes to improve the reproducibility of the work that we publish. This form provides structure for consistency and transparency in reporting. For further information on Nature Portfolio policies, see our Editorial Policies and the Editorial Policy Checklist.

Statistics

For all statistical analyses, confirm that the following items are present in the figure legend, table legend, main text, or Methods section.

n/a	Confirmed	
☐	☒	The exact sample size (*n*) for each experimental group/condition, given as a discrete number and unit of measurement
☐	☒	A statement on whether measurements were taken from distinct samples or whether the same sample was measured repeatedly
☐	☒	The statistical test(s) used AND whether they are one- or two-sided<br>*Only common tests should be described solely by name; describe more complex techniques in the Methods section.*
☒	☐	A description of all covariates tested
☒	☐	A description of any assumptions or corrections, such as tests of normality and adjustment for multiple comparisons
☐	☒	A full description of the statistical parameters including central tendency (e.g. means) or other basic estimates (e.g. regression coefficient) AND variation (e.g. standard deviation) or associated estimates of uncertainty (e.g. confidence intervals)
☐	☒	For null hypothesis testing, the test statistic (e.g. *F*, *t*, *r*) with confidence intervals, effect sizes, degrees of freedom and *P* value noted<br>*Give P values as exact values whenever suitable.*
☒	☐	For Bayesian analysis, information on the choice of priors and Markov chain Monte Carlo settings
☒	☐	For hierarchical and complex designs, identification of the appropriate level for tests and full reporting of outcomes
☐	☒	Estimates of effect sizes (e.g. Cohen's *d*, Pearson's *r*), indicating how they were calculated

Our web collection on statistics for biologists contains articles on many of the points above.

Software and code

Policy information about availability of computer code

Data collection	All softwares used for data collection are publicly or commercially available:<br>Sagecreation MiniChemi 500 system for Western Blot;<br>BIO RAD CFX3.1 manager for qPCR;<br>Tecan SparkControl V1.8 for absorbance;<br>ZEN 2010 for Immunofluorescence images;<br>Leica LAS X (3.0.2.16120) Navigator for IHC images;<br>NIS-Elements 4.2.0 for soft agar images;<br>The mRNA levels of TMEM25 in breast cancer and colorectal cancer samples were from UALCAN platform.<br>Kaplan-Meier survival curves of colorectal cancer patients and breast cancer patients were from UCSC Xena platform.
Data analysis	All softwares used for data analysis are publicly or commercially available:<br>Statistical analyses were performed on GraphPad Prism 8.0.2;<br>Western Blot analyses were performed on Lane 1D 5.1.0.0 software.

For manuscripts utilizing custom algorithms or software that are central to the research but not yet described in published literature, software must be made available to editors and reviewers. We strongly encourage code deposition in a community repository (e.g. GitHub). See the Nature Portfolio guidelines for submitting code & software for further information.

Data

Policy information about availability of data

All manuscripts must include a data availability statement. This statement should provide the following information, where applicable:

- Accession codes, unique identifiers, or web links for publicly available datasets
- A description of any restrictions on data availability
- For clinical datasets or third party data, please ensure that the statement adheres to our policy

All data supporting the findings of this study are available within the article and its Supplementary Information files.
The datasets from the cancer genome atlas (TCGA) (https://www.cancer.gov/ccg/research/genome-sequencing/tcga) were analyzed using the UALCAN platform (http://ualcan.path.uab.edu/analysis.html).
UCSC Xena platform (https://xena.ucsc.edu) was used for survival analysis in breast cancer and colorectal cancer patients.

Human research participants

Policy information about studies involving human research participants and Sex and Gender in Research.

Reporting on sex and gender	Female
Population characteristics	28 primary human breast cancer tissue samples and corresponding adjacent normal tissues were from female TNBC patients. The information of patients are summarized in Supplementary Table1.
Recruitment	All samples taken after surgery were stocked in liquid nitrogen for further analysis.
Ethics oversight	The study was in accordance with research ethics board approval from Xiamen University and the Affiliate Hospitals. Informed consent was obtained from all patients.

Note that full information on the approval of the study protocol must also be provided in the manuscript.

Field-specific reporting

Please select the one below that is the best fit for your research. If you are not sure, read the appropriate sections before making your selection.

☒ Life sciences ☐ Behavioural & social sciences ☐ Ecological, evolutionary & environmental sciences

For a reference copy of the document with all sections, see nature.com/documents/nr-reporting-summary-flat.pdf

Life sciences study design

All studies must disclose on these points even when the disclosure is negative.

Sample size	For each treatment in our manuscript, a sample size was determined accordingly to previous published study and experimental knowledge. Sample sizes for mouse experiments were determined based on previous published study (Lintao Song Nature communications 2020, Taoling Zeng Cell reports 2014, Qingang Wu Science Advances 2020). For in vitro experiments, sample sizes were determined based on previous published study in the lab (Taoling Zeng Cell reports 2014, Mingdong Liu Nature communications 2018, Hong-Rui Wang Science 2003).
Data exclusions	No data were excluded from the analyses.
Replication	All experiments were repeated at least three times and similar results were obtained.
Randomization	All experimental groups for animal studies were at similar age and randomly assigned to different treatments. For cell experiments, cells of each genotype were parallel seeded and randomly assigned to different treatments.
Blinding	The investigators were blind during data collection and analysis.

Behavioural & social sciences study design

All studies must disclose on these points even when the disclosure is negative.

Study description	*Briefly describe the study type including whether data are quantitative, qualitative, or mixed-methods (e.g. qualitative cross-sectional, quantitative experimental, mixed-methods case study).*

Research sample	*State the research sample (e.g. Harvard university undergraduates, villagers in rural India) and provide relevant demographic information (e.g. age, sex) and indicate whether the sample is representative. Provide a rationale for the study sample chosen. For studies involving existing datasets, please describe the dataset and source.*
Sampling strategy	*Describe the sampling procedure (e.g. random, snowball, stratified, convenience). Describe the statistical methods that were used to predetermine sample size OR if no sample-size calculation was performed, describe how sample sizes were chosen and provide a rationale for why these sample sizes are sufficient. For qualitative data, please indicate whether data saturation was considered, and what criteria were used to decide that no further sampling was needed.*
Data collection	*Provide details about the data collection procedure, including the instruments or devices used to record the data (e.g. pen and paper, computer, eye tracker, video or audio equipment) whether anyone was present besides the participant(s) and the researcher, and whether the researcher was blind to experimental condition and/or the study hypothesis during data collection.*
Timing	*Indicate the start and stop dates of data collection. If there is a gap between collection periods, state the dates for each sample cohort.*
Data exclusions	*If no data were excluded from the analyses, state so OR if data were excluded, provide the exact number of exclusions and the rationale behind them, indicating whether exclusion criteria were pre-established.*
Non-participation	*State how many participants dropped out/declined participation and the reason(s) given OR provide response rate OR state that no participants dropped out/declined participation.*
Randomization	*If participants were not allocated into experimental groups, state so OR describe how participants were allocated to groups, and if allocation was not random, describe how covariates were controlled.*

Ecological, evolutionary & environmental sciences study design

All studies must disclose on these points even when the disclosure is negative.

Study description	*Briefly describe the study. For quantitative data include treatment factors and interactions, design structure (e.g. factorial, nested, hierarchical), nature and number of experimental units and replicates.*
Research sample	*Describe the research sample (e.g. a group of tagged Passer domesticus, all Stenocereus thurberi within Organ Pipe Cactus National Monument), and provide a rationale for the sample choice. When relevant, describe the organism taxa, source, sex, age range and any manipulations. State what population the sample is meant to represent when applicable. For studies involving existing datasets, describe the data and its source.*
Sampling strategy	*Note the sampling procedure. Describe the statistical methods that were used to predetermine sample size OR if no sample-size calculation was performed, describe how sample sizes were chosen and provide a rationale for why these sample sizes are sufficient.*
Data collection	*Describe the data collection procedure, including who recorded the data and how.*
Timing and spatial scale	*Indicate the start and stop dates of data collection, noting the frequency and periodicity of sampling and providing a rationale for these choices. If there is a gap between collection periods, state the dates for each sample cohort. Specify the spatial scale from which the data are taken*
Data exclusions	*If no data were excluded from the analyses, state so OR if data were excluded, describe the exclusions and the rationale behind them, indicating whether exclusion criteria were pre-established.*
Reproducibility	*Describe the measures taken to verify the reproducibility of experimental findings. For each experiment, note whether any attempts to repeat the experiment failed OR state that all attempts to repeat the experiment were successful.*
Randomization	*Describe how samples/organisms/participants were allocated into groups. If allocation was not random, describe how covariates were controlled. If this is not relevant to your study, explain why.*
Blinding	*Describe the extent of blinding used during data acquisition and analysis. If blinding was not possible, describe why OR explain why blinding was not relevant to your study.*

Did the study involve field work? ☐ Yes ☐ No

Field work, collection and transport

Field conditions	*Describe the study conditions for field work, providing relevant parameters (e.g. temperature, rainfall).*
Location	*State the location of the sampling or experiment, providing relevant parameters (e.g. latitude and longitude, elevation, water depth).*
Access & import/export	*Describe the efforts you have made to access habitats and to collect and import/export your samples in a responsible manner and in*

Access & import/export	compliance with local, national and international laws, noting any permits that were obtained (give the name of the issuing authority, the date of issue, and any identifying information).
Disturbance	Describe any disturbance caused by the study and how it was minimized.

Reporting for specific materials, systems and methods

We require information from authors about some types of materials, experimental systems and methods used in many studies. Here, indicate whether each material, system or method listed is relevant to your study. If you are not sure if a list item applies to your research, read the appropriate section before selecting a response.

Materials & experimental systems

n/a	Involved in the study
☐	☒ Antibodies
☐	☒ Eukaryotic cell lines
☒	☐ Palaeontology and archaeology
☐	☒ Animals and other organisms
☒	☐ Clinical data
☒	☐ Dual use research of concern

Methods

n/a	Involved in the study
☒	☐ ChIP-seq
☒	☐ Flow cytometry
☒	☐ MRI-based neuroimaging

Antibodies

Antibodies used

β-Actin Antibody (C4) mouse monoclonal IgG Santa Cruz Biotechnology cat# sc-47778 WB
GST Antibody (B-14) mouse monoclonal IgG Santa Cruz Biotechnology cat# sc-138 WB
GFP Antibody (B-2) mouse monoclonal IgG Santa Cruz Biotechnology cat# sc-9996 WB
β-Tubulin (TUB 2.1, monoclonal) Antibody mouse monoclonal IgG Sigma-Aldrich cat#T4026 WB
FLAG Antibody (M2) mouse monoclonal IgG Sigma-Aldrich cat# F1804 WB IP
TMEM25 Antibody rabbit polyclonal IgG Sigma-Aldrich cat# HPA012163 WB IP
Ki67 Antibody (D3B5) rabbit monoclonal IgG Cell Signaling Technology cat# 12202 IHC
Phospho-STAT3 (Y705)(D3A7) rabbit monoclonal IgG Cell Signaling Technology cat# 9145 WB IHC
STAT3 (D3Z2G) rabbit monoclonal IgG Cell Signaling Technology cat# 12640 WB
Phospho-EGFR (Y1068) (D7A5) rabbit monoclonal IgG Cell Signaling Technology cat# 3777 WB
Phospho-EGFR (Y1086) rabbit polyclonal IgG Cell Signaling Technology cat# 2220 WB
EGFR (D38B1) rabbit monoclonal IgG Cell Signaling Technology cat# 4267 WB IP IF
Phospho-STAT5 (Y694) rabbit polyclonal IgG Cell Signaling Technology cat# 9351 WB
STAT5 (D2O6Y) rabbit monoclonal IgG Cell Signaling Technology cat# 94205 WB
Phospho-AKT (S473) rabbit polyclonal IgG Cell Signaling Technology cat# 9271 WB
AKT (pan) (C67E7) rabbit monoclonal IgG Cell Signaling Technology cat# 4691 WB
Phospho-ERK1/2 (Thr202/Tyr204) (D13.14.4E) rabbit monoclonal IgG Cell Signaling Technology cat# 4370 WB
ERK1/2 rabbit polyclonal IgG Cell Signaling Technology cat# 9102 WB
JAK1 (6G4) rabbit monoclonal IgG Cell Signaling Technology cat# 3344 WB
JAK2 (D2E12) rabbit monoclonal IgG Cell Signaling Technology cat# 3230 WB
SRC (36D10) rabbit monoclonal IgG Cell Signaling Technology cat# 2109 WB
HA (3F10) rat monoclonal antibody Roche cat# 11867431001 WB
HRP anti-phosphotyrosine (pY20) antibody abcam cat# ab16389
Goat anti-Rabbit IgG (H+L) Secondary Antibody, HRP ThermoFisher cat# 31460
Goat anti-Mouse IgG (H+L) Secondary Antibody, HRP ThermoFisher cat# 31430
Goat anti-Rat IgG (H+L) Secondary Antibody, HRP ThermoFisher cat# 31470
Alexa Fluor 555 donkey anti-rabbit ThermoFisher cat# A31572

Validation

β-Actin Antibody (C4) mouse monoclonal Santa Cruz Biotechnology cat# sc-47778:
mouse, rat, human, avian; WB (1:2000)
western blot analysis of extracts from human Hela cells and mouse NIH/3T3 cells (instruction from manufacturer's website).
GST Antibody (B-14) mouse monoclonal Santa Cruz Biotechnology cat# sc-138:
human recombinant and Schistosoma japonicum recombinant GST fusion proteins; WB (1:2000)
western blot analysis of human recombinant and Schistosoma japonicum recombinant GST fusion proteins (instruction from manufacturer's website).
GFP Antibody (B-2) mouse monoclonal IgG Santa Cruz Biotechnology cat# sc-9996:
GFP and GFP mutant fusion proteins; WB (1:2000), IP (1:200)
western blot analysis of GFP expression in human recombinant GFP fusion protein (instruction from manufacturer's website).
β-Tubulin (TUB 2.1, monoclonal) Antibody mouse monoclonal IgG Sigma-Aldrich cat#T4026:
human, rat, frog, moth, mouse, plant, rabbit; WB (1:2000)
western blot analysis of extracts from human Hela cells (instruction from manufacturer's website).
FLAG Antibody (M2) mouse monoclonal IgG Sigma-Aldrich cat# F1804:
All; IF (1:100),IP (1:200),WB (1:2000)
this antibody has been validated in human Hela cells and mouse NIH3T3 cells (instruction from manufacturer's website).
TMEM25 Antibody rabbit polyclonal IgG Sigma-Aldrich cat# HPA012163:

human, mouse; WB (1:1000), IP (1:100)
the antibody specificity was tested by TMEM25 knockout human MDA-MB-231 cells and mouse 4T1 cells (our manuscript Fig.5a-b).
Ki67 Antibody (D3B5) rabbit monoclonal IgG Cell Signaling Technology cat# 12202:
human, mouse, rat; IHC (1:100)
immunohistochemical analysis of paraffin-embedded mouse colon (instruction from manufacturer's website).
Phospho-STAT3 (Y705) (D3A7) rabbit monoclonal IgG Cell Signaling Technology cat# 9145:
human, mouse, rat, monkey; WB (1:2000), IHC (1:100)
western blot analysis of extracts from IFN-alpha treated human Jurkat cells and HeLa cells, as well as
EGF treated A431 cells. Immunohistochemical analysis of paraffin-embedded mouse colon (instruction from manufacturer's website).
STAT3 (D3Z2G) rabbit monoclonal IgG Cell Signaling Technology cat# 12640:
human, mouse, rat, monkey; WB (1:2000)
Western blot analysis of extracts from human PC3 cells (instruction from manufacturer's website).
Phospho-EGFR (Y1068) (D7A5) rabbit monoclonal IgG Cell Signaling Technology cat# 3777:
human, mouse, rat, monkey; WB (1:2000)
Western blot analysis of extracts of untreated or EGF-stimulated human BxPC-3 cells (instruction from manufacturer's website).
Phospho-EGFR (Y1086) rabbit polyclonal IgG Cell Signaling Technology cat# 2220:
human, mouse; WB (1:2000)
Western blot analysis of cell extracts from untreated or EGF-treated human A431 cells (instruction from manufacturer's website).
EGFR (D38B1) rabbit monoclonal IgG Cell Signaling Technology cat# 4267:
human, mouse, monkey; WB (1:2000), IP (1:100), IF (1:100)
Western blot analysis of extracts from control human Hela cells, or EGFR knockout Hela cells.
Immunoprecipitation of EGF Receptor from HeLa cell extracts. Confocal immunofluorescent analysis
of untreated or treated with human epidermal growth factor human A549 cells. (instruction from manufacturer's website)
Phospho-STAT5 (Y694) rabbit polyclonal IgG Cell Signaling Technology cat# 9351:
human, mouse; WB (1:2000)
Western blot analysis of extracts from human TF-1 cells, treated with GM-CSF (25 ng/ml) for 15 min, 30 min,1 h, 2 h, 4 h, 8 h, 24 h (instruction from manufacturer's website).
STAT5 (D2O6Y) rabbit monoclonal IgG Cell Signaling Technology cat# 94205:
human, mouse, rat; WB (1:2000)
Western blot analysis of extracts from human K562 cells (instruction from manufacturer's website).
Phospho-AKT (S473) rabbit polyclonal IgG Cell Signaling Technology cat# 9271:
human, mouse, rat; WB (1:2000)
Western blot analysis of extracts from mouse NIH/3T3 cells, untreated or treated with PDGF, wortmannin, LY294002, rapamycin or PD98059 (instruction from manufacturer's website).
AKT (pan) (C67E7) rabbit monoclonal IgG Cell Signaling Technology cat# 4691:
human, mouse, rat; WB (1:2000)
Western blot analysis of recombinant Akt1, Akt2 and Akt3 proteins, and extracts from human Hela cells and mouseNIH3T3 cells (instruction from manufacturer's website).
Phospho-ERK1/2 (Thr202/Tyr204) (D13.14.4E) rabbit monoclonal IgG Cell Signaling Technology cat# 4370:
human, mouse, rat; WB (1:2000)
Western blot analysis of extracts from human 293 cells and NIH3T3 cells (instruction from manufacturer's website).
ERK1/2 rabbit polyclonal IgG Cell Signaling Technology cat# 9102:
human, mouse, rat; WB (1:2000)
Western blot analysis of extracts from human Hela cells (instruction from manufacturer's website).
JAK1 (6G4) rabbit monoclonal IgG Cell Signaling Technology cat# 3344:
human, mouse, rat; WB (1:2000)
Western blot analysis of extracts from human ACHN, SR cells, mouse CTLL-2 cells and rat PC12 cell lines
(instruction from manufacturer's website) and this antibody was tested in JAK1 knockdown human MDA-MB-231cells (our manuscript Fig.4a).
JAK2 (D2E12) rabbit monoclonal IgG Cell Signaling Technology cat# 3230:
human, mouse, rat; WB (1:2000)
Western blot analysis of extracts from human K-562, THP-1, TF-1 and mouse BaF3 cell lines
(instruction from manufacturer's website) and this antibody was tested in JAK2 knockdown cells (our manuscript Fig.4a).
SRC (36D10) rabbit monoclonal IgG Cell Signaling Technology cat# 2109:
human, mouse, rat; WB (1:2000)
Western blot analysis of extracts from human A-431 and mouse C2C12 cells (instruction from manufacturer's website)
and this antibody was tested in JAK2 knockdown cells (our manuscript Fig.4a).
HA (3F10) rat monoclonal antibody Roche cat# 11867431001:
human, mouse, rat; WB (1:2000)
HA (12CA5) mouse monoclonal antibody Roche cat# 11666606001:
human, mouse, rat; IP (1:200)
Use anti-HA High Affinity for the detection of native influenza hemagglutinin protein and recombinant proteins
that contain the HA epitope using in Dot blots, ELISA, Immunocytochemistry, Immunoprecipitation,
western blots (instruction from manufacturer's website).
HRP anti-phosphotyrosine (pY20) antibody abcam cat# ab16389:
human, mouse; WB (1:2000)
Western blot analysis of mouse NIH 3T3 whole cell lysate (instruction from manufacturer's website).
Goat anti-Rabbit IgG (H+L) Secondary Antibody, HRP ThermoFisher cat# 31460
Western blot analysis was performed on whole cell extracts of human A549 and Hep G2 cells. The blots were probed
with Anti-CacyBP Rabbit Polyclonal Antibody and detected by chemiluminescence using Goat anti-Rabbit IgG (H+L) Secondary Antibody,
HRP conjugate (instruction from manufacturer's website).
Goat anti-Mouse IgG (H+L) Secondary Antibody, HRP ThermoFisher cat# 31430:
Western blot analysis was performed on whole cell extracts of human K-562 and U87-MG. The blots were probed with
Anti-SOD2 Mouse Monoclonal Antibody and detected by chemiluminescence using Goat anti-Mouse IgG (H+L)

Secondary Antibody, HRP conjugate (instruction from manufacturer's website).
Goat anti-Rat IgG (H+L) Secondary Antibody, HRP ThermoFisher cat# 31470:
Western blot analysis was performed on whole cell extracts of mouse F9 cells and human A549 cells. The blots were probed with Anti-alpha Tubulin Antibody (YL1/2) Rat Monoclonal Antibody and detected by chemiluminescence using Goat anti-Rat IgG (H+L) Secondary Antibody, HRP conjugate (instruction from manufacturer's website).
Alexa Fluor 555 donkey anti-rabbit ThermoFisher cat# A31572:
Secondary Antibody Alexa Fluor 555 conjugate was performed using human HeLa cells stained with alpha Tubulin Rabbit Polyclonal Antibody (instruction from manufacturer's website).

Eukaryotic cell lines

Policy information about cell lines and Sex and Gender in Research

Cell line source(s)	HEK293T, MDA-MB-231, MCF7, BT549, HCC1937, and 4T1 were obtained from ATCC. TMEM25+/+ and TMEM25-/- MEF cells were isolated from female TMEM25+/+ and female TMEM25-/- mice, respectively. TMEM25+/+ and TMEM25-/- tumor cells were isolated from female PyMT;TMEM25+/+ and female PyMT;TMEM25-/- mice, respectively.
Authentication	HEK293T, MCF7, MDA-MB-231, BT549, HCC1937, and 4T1 were were authenticated with morphology, karyotyping, and PCR based approaches by ATCC.
Mycoplasma contamination	All cells were tested for mycoplasma contamination and were found to be negative.
Commonly misidentified lines (See ICLAC register)	No commonly misidentified cell lines were used in this study.

Palaeontology and Archaeology

Specimen provenance	*Provide provenance information for specimens and describe permits that were obtained for the work (including the name of the issuing authority, the date of issue, and any identifying information). Permits should encompass collection and, where applicable, export.*
Specimen deposition	*Indicate where the specimens have been deposited to permit free access by other researchers.*

Dating methods | *If new dates are provided, describe how they were obtained (e.g. collection, storage, sample pretreatment and measurement), where they were obtained (i.e. lab name), the calibration program and the protocol for quality assurance OR state that no new dates are provided.*

☐ Tick this box to confirm that the raw and calibrated dates are available in the paper or in Supplementary Information.

Ethics oversight | *Identify the organization(s) that approved or provided guidance on the study protocol, OR state that no ethical approval or guidance was required and explain why not.*

Note that full information on the approval of the study protocol must also be provided in the manuscript.

Animals and other research organisms

Policy information about studies involving animals; ARRIVE guidelines recommended for reporting animal research, and Sex and Gender in Research

Laboratory animals	4-6 weeks old Female BALB/c mice were used for 4T1 cells injection.<br>4-6 weeks old Female nude mice were used for MDA-MB-231 cells injection.<br>8-10 weeks old Female MMTV-PyMT mice, 4-6 weeks old Female C57BL/6 mice and 6-8 weeks old male EIIA mice were used in this study.
Wild animals	No wild animal was used in this study.
Reporting on sex	Female
Field-collected samples	The study did not involve field-collected sample.
Ethics oversight	All experiments conducted in this study were approved by Institutional Animal Care and Use Committee of Xiamen University.

Note that full information on the approval of the study protocol must also be provided in the manuscript.

Clinical data

Policy information about clinical studies
All manuscripts should comply with the ICMJE guidelines for publication of clinical research and a completed CONSORT checklist must be included with all submissions.

Clinical trial registration	*Provide the trial registration number from ClinicalTrials.gov or an equivalent agency.*
Study protocol	*Note where the full trial protocol can be accessed OR if not available, explain why.*
Data collection	*Describe the settings and locales of data collection, noting the time periods of recruitment and data collection.*
Outcomes	*Describe how you pre-defined primary and secondary outcome measures and how you assessed these measures.*

Dual use research of concern

Policy information about dual use research of concern

Hazards

Could the accidental, deliberate or reckless misuse of agents or technologies generated in the work, or the application of information presented in the manuscript, pose a threat to:

No	Yes	
☒	☐	Public health
☒	☐	National security
☒	☐	Crops and/or livestock
☒	☐	Ecosystems
☒	☐	Any other significant area

Experiments of concern

Does the work involve any of these experiments of concern:

No	Yes	
☒	☐	Demonstrate how to render a vaccine ineffective
☒	☐	Confer resistance to therapeutically useful antibiotics or antiviral agents
☒	☐	Enhance the virulence of a pathogen or render a nonpathogen virulent
☒	☐	Increase transmissibility of a pathogen
☒	☐	Alter the host range of a pathogen
☒	☐	Enable evasion of diagnostic/detection modalities
☒	☐	Enable the weaponization of a biological agent or toxin
☒	☐	Any other potentially harmful combination of experiments and agents

ChIP-seq

Data deposition

☐ Confirm that both raw and final processed data have been deposited in a public database such as GEO.

☐ Confirm that you have deposited or provided access to graph files (e.g. BED files) for the called peaks.

Data access links<br>*May remain private before publication.*	*For "Initial submission" or "Revised version" documents, provide reviewer access links. For your "Final submission" document, provide a link to the deposited data.*
Files in database submission	*Provide a list of all files available in the database submission.*
Genome browser session<br>(e.g. UCSC)	*Provide a link to an anonymized genome browser session for "Initial submission" and "Revised version" documents only, to enable peer review. Write "no longer applicable" for "Final submission" documents.*

Methodology

Replicates	*Describe the experimental replicates, specifying number, type and replicate agreement.*
Sequencing depth	*Describe the sequencing depth for each experiment, providing the total number of reads, uniquely mapped reads, length of reads and whether they were paired- or single-end.*
Antibodies	*Describe the antibodies used for the ChIP-seq experiments; as applicable, provide supplier name, catalog number, clone name, and lot number.*
Peak calling parameters	*Specify the command line program and parameters used for read mapping and peak calling, including the ChIP, control and index files used.*
Data quality	*Describe the methods used to ensure data quality in full detail, including how many peaks are at FDR 5% and above 5-fold enrichment.*
Software	*Describe the software used to collect and analyze the ChIP-seq data. For custom code that has been deposited into a community repository, provide accession details.*

Flow Cytometry

Plots

Confirm that:

☐ The axis labels state the marker and fluorochrome used (e.g. CD4-FITC).

☐ The axis scales are clearly visible. Include numbers along axes only for bottom left plot of group (a 'group' is an analysis of identical markers).

☐ All plots are contour plots with outliers or pseudocolor plots.

☐ A numerical value for number of cells or percentage (with statistics) is provided.

Methodology

Sample preparation	*Describe the sample preparation, detailing the biological source of the cells and any tissue processing steps used.*
Instrument	*Identify the instrument used for data collection, specifying make and model number.*

Software	*Describe the software used to collect and analyze the flow cytometry data. For custom code that has been deposited into a community repository, provide accession details.*
Cell population abundance	*Describe the abundance of the relevant cell populations within post-sort fractions, providing details on the purity of the samples and how it was determined.*
Gating strategy	*Describe the gating strategy used for all relevant experiments, specifying the preliminary FSC/SSC gates of the starting cell population, indicating where boundaries between "positive" and "negative" staining cell populations are defined.*

☐ Tick this box to confirm that a figure exemplifying the gating strategy is provided in the Supplementary Information.

Magnetic resonance imaging

Experimental design

Design type	*Indicate task or resting state; event-related or block design.*
Design specifications	*Specify the number of blocks, trials or experimental units per session and/or subject, and specify the length of each trial or block (if trials are blocked) and interval between trials.*
Behavioral performance measures	*State number and/or type of variables recorded (e.g. correct button press, response time) and what statistics were used to establish that the subjects were performing the task as expected (e.g. mean, range, and/or standard deviation across subjects).*

Acquisition

Imaging type(s)	*Specify: functional, structural, diffusion, perfusion.*
Field strength	*Specify in Tesla*
Sequence & imaging parameters	*Specify the pulse sequence type (gradient echo, spin echo, etc.), imaging type (EPI, spiral, etc.), field of view, matrix size, slice thickness, orientation and TE/TR/flip angle.*
Area of acquisition	*State whether a whole brain scan was used OR define the area of acquisition, describing how the region was determined.*

Diffusion MRI ☐ Used ☐ Not used

Preprocessing

Preprocessing software	*Provide detail on software version and revision number and on specific parameters (model/functions, brain extraction, segmentation, smoothing kernel size, etc.).*
Normalization	*If data were normalized/standardized, describe the approach(es): specify linear or non-linear and define image types used for transformation OR indicate that data were not normalized and explain rationale for lack of normalization.*
Normalization template	*Describe the template used for normalization/transformation, specifying subject space or group standardized space (e.g. original Talairach, MNI305, ICBM152) OR indicate that the data were not normalized.*
Noise and artifact removal	*Describe your procedure(s) for artifact and structured noise removal, specifying motion parameters, tissue signals and physiological signals (heart rate, respiration).*
Volume censoring	*Define your software and/or method and criteria for volume censoring, and state the extent of such censoring.*

Statistical modeling & inference

Model type and settings	*Specify type (mass univariate, multivariate, RSA, predictive, etc.) and describe essential details of the model at the first and second levels (e.g. fixed, random or mixed effects; drift or auto-correlation).*
Effect(s) tested	*Define precise effect in terms of the task or stimulus conditions instead of psychological concepts and indicate whether ANOVA or factorial designs were used.*

Specify type of analysis: ☐ Whole brain ☐ ROI-based ☐ Both

Statistic type for inference (See Eklund et al. 2016)	*Specify voxel-wise or cluster-wise and report all relevant parameters for cluster-wise methods.*
Correction	*Describe the type of correction and how it is obtained for multiple comparisons (e.g. FWE, FDR, permutation or Monte Carlo).*

Models & analysis

n/a	Involved in the study
☐	☐ Functional and/or effective connectivity
☐	☐ Graph analysis
☐	☐ Multivariate modeling or predictive analysis

Functional and/or effective connectivity	*Report the measures of dependence used and the model details (e.g. Pearson correlation, partial correlation, mutual information).*
Graph analysis	*Report the dependent variable and connectivity measure, specifying weighted graph or binarized graph, subject- or group-level, and the global and/or node summaries used (e.g. clustering coefficient, efficiency, etc.).*
Multivariate modeling and predictive analysis	*Specify independent variables, features extraction and dimension reduction, model, training and evaluation metrics.*
